# Synthesis and Performance of 6FDA-Based Polyimide-Ionenes and Composites with Ionic Liquids as Gas Separation Membranes

**DOI:** 10.3390/membranes9070079

**Published:** 2019-07-03

**Authors:** Kathryn E. O’Harra, Irshad Kammakakam, Emily M. Devriese, Danielle M. Noll, Jason E. Bara, Enrique M. Jackson

**Affiliations:** 1 Department of Chemical & Biological Engineering, University of Alabama, Tuscaloosa, AL 35487-0203, USA; 2 NASA Marshall Space Flight Center, Huntsville, AL 35812, USA

**Keywords:** 6FDA polyimides, ionenes, Hybrid Membranes, CO_2_ Separation

## Abstract

Three new isomeric 6FDA-based polyimide-ionenes, with imidazolium moieties and varying regiochemistry (*para-, meta-, and ortho-* connectivity), and composites with three different ionic liquids (ILs) have been developed as gas separation membranes. The structural-property relationships and gas separation behaviors of the newly developed 6FDA polyimide-ionene + IL composites have been extensively studied. All the 6FDA-based polyimide-ionenes exhibited good compatibility with the ILs and produced homogeneous hybrid membranes with the high thermal stability of ~380 °C. Particularly, [6FDA I4A pXy][Tf_2_N] ionene + IL hybrids having [C_4_mim][Tf_2_N] and [Bnmim][Tf_2_N] ILs offered mechanically stable matrixes with high CO_2_ affinity. The permeability of CO_2_ was increased by factors of 2 and 3 for C_4_mim and Bnmim hybrids (2.15 to 6.32 barrers), respectively, compared to the neat [6FDA I4A pXy][Tf_2_N] without sacrificing their permselectivity for CO_2_/CH_4_ and CO_2_/N_2_ gas pairs.

## 1. Introduction

Polyimides (PIs) have been extensively considered as materials for gas separation membranes because of their excellent mechanical and thermal stability as well as their intrinsic separation properties, particularly for CO_2_/CH_4_ and CO_2_/N_2_ separations [1,2,3]. Furthermore, the synthesis and functionalization of PIs are neither problematic nor expensive, and it is easy to fabricate ultra-thin membranes using current commercial fabrication processes. The gas separation performance of the polymeric membranes is directly related to the chemical structure of the polymer and morphology. Therefore, research on the structures and properties of polymers is of great interest and an enduring challenge in the development of gas separation membranes. Research into the structure-property relationships of PIs with the aim of developing enhanced gas separation application membranes has mainly focused on (1) the inclusion of flexible or contorted substituent groups into the PI backbone to enhance processability [4,5,6], (2) the incorporation of bulky and polar groups in order to improve the permeability/selectivity tradeoffs [7,8], (3) the enhancement of the inter-chain packing density and the dimensions of free volume elements [9,10], and (4) the improvement of the thermal and mechanical resistance of such membranes to harsh environments [11,12,13].

Among the various research reports on PI membrane materials to explore the relationship between the chemical structures and the gas transport properties, fluorinated dianhydride, 4,4′-(hexafluoroisopropylidene)-diphthalic anhydride (commonly abbreviated as 6FDA) based PIs have recently dominated the literature and are observed to have much higher CO_2_ permeabilities with reasonable selectivities [11,14]. The 6FDA-PI contains -CF_3_ groups which restrict the torsional motion of the neighboring phenyl rings, and thus, prevents efficient chain packing, which yields high fractional free volume (FFV) in the polymeric matrix and enhances the permeability. On the other hand, there have been several attempts to improve the selectivity of such PIs by introducing CO_2_-philic species, such as ionic liquids (ILs) [15,16], amines [17], or poly(ethylene oxide)s [18].

ILs have recently emerged as promising CO_2_ separation materials due to their CO_2_ affinity and high CO_2_ gas selectivity over N_2_ and CH_4_ [19,20]. Polymeric membranes either containing or built from ILs offer virtually endless possibilities for the design of materials for enhanced gas separation applications. Among the cations commonly used to form ILs or Poly(IL)s, including phosphonium [21], ammonium [22] and pyridinium [23], the imidazolium cations have garnered the more interest due to their versatile nature and tunable structures [24,25]. Furthermore, it is highly feasible to graft pendant imidazolium cations to most conventional polymers such as poly(ether sulfone)s [26], poly(ether ketone)s [27], and polyimides [8,28]. Nonetheless, there have been just a handful of reports considering those pendant imidazolium polymers in CO_2_ separation membranes, especially from a PI backbone. It also should be noted that pendant imidazolium groups can be cleaved from polymer backbones at elevated temperatures (nearly 250 °C) [29].

Ionenes are charged polymers in which the ionic moieties are contained directly within the polymer backbone rather than as pendants. Ionenes are also of growing interest as a material platform for gas separation, as well as many other applications, including electrolytes for fuel cells and Li-ion batteries [30,31,32]. With a polycationic structure (mainly as a quaternary ammonium form), ionenes function as a robust matrix that yields high thermal stability and provides Coulombic interactions between polymer chains, retaining the properties of ILs. Recently, we have focused on the design and synthesis of various bis(imidazole) monomers containing aromatic imide or amide linkages that can yield thermally robust imidazolium ionenes [33,34]. These “high-performance” ionenes can be prepared via simple synthetic strategies without significant additional production costs and are well-suited to separate CO_2_ from light gases such as N_2_ and CH_4_ with high selectivity and durability [34].

**Scheme 1:**
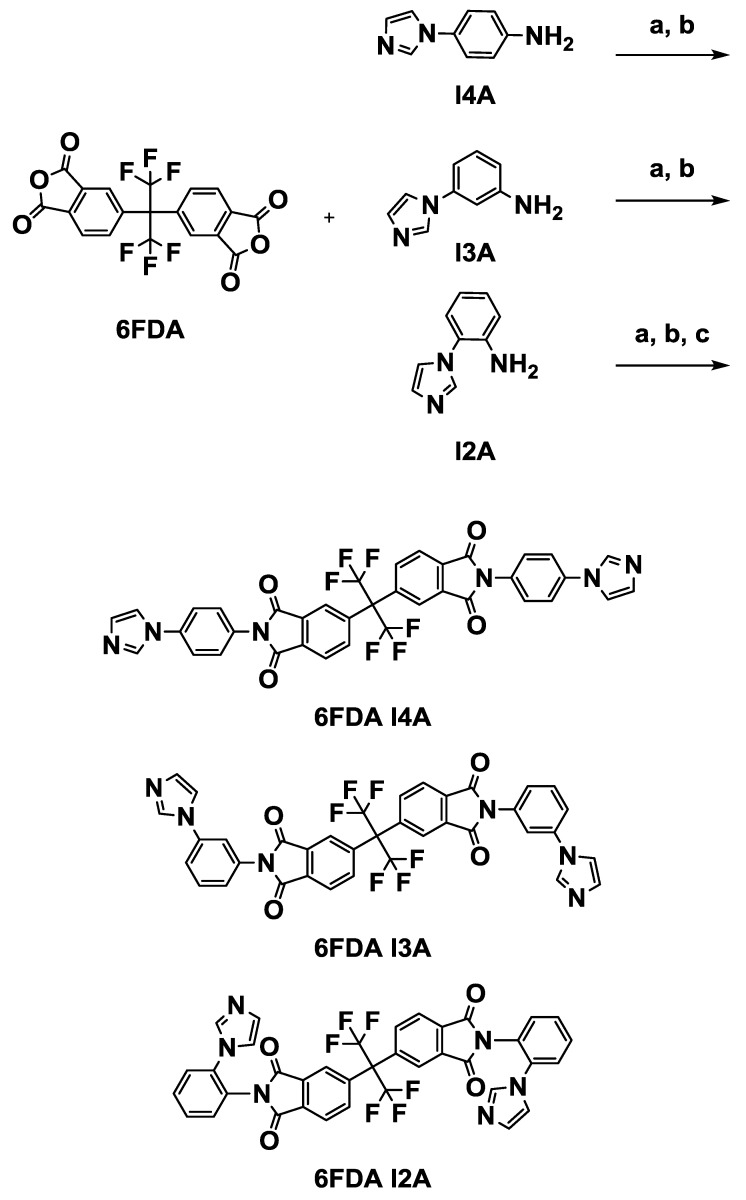
Synthesis of aromatic, fluorinated dianhydride, 4,4′-(hexafluoroisopropylidene)-diphthalic anhydride (6FDA)-based imide monomers. a: DMF (5 °C, 1 h; RT, 24 h); b: Toluene (125 °C, 24 h); c: Ac_2_O (70 °C, 16 h).

**Scheme 2:**
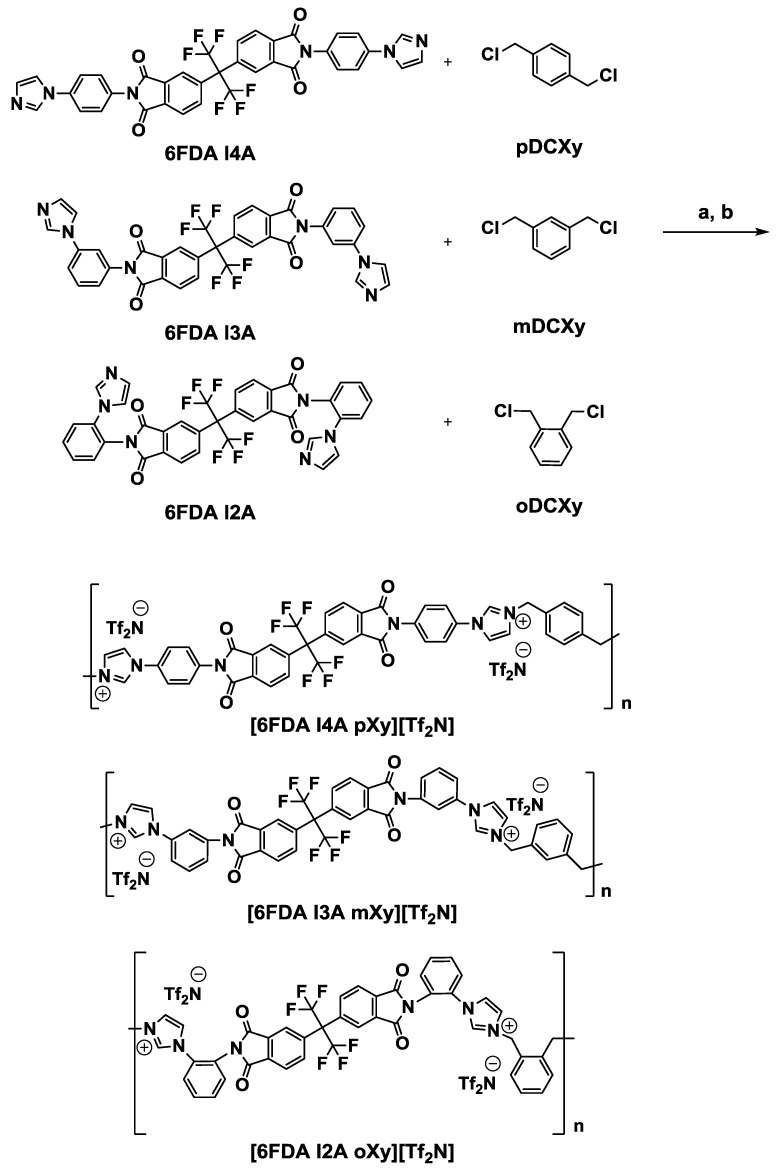
Synthesis of imidazolium polyimide-ionene derivatives. a: DMF (120 °C, 24 h); b: H_2_O, LiTf_2_N (RT, 24 h).

Herein, we report the design, synthesis, and gas separation performance of a series of three 6FDA-based PI-ionenes and composites with three imidazolium ILs: [C_2_mim][Tf_2_N], [C_4_mim][Tf_2_N], and [Bnmim][Tf_2_N]. As depicted in Scheme 1, three imide-functionalized monomers were prepared via the reaction of 6FDA with imidazole-aniline molecules connected at *para-, meta-*, or *ortho* positions. A step-growth polycondensation of these three 6FDA-imidazole monomers with a stoichiometric equivalent of an α,α’-dichloroxylene (*para-, meta-*, *and ortho*) via Menshutkin reactions yielded corresponding 6FDA-based PI-ionenes (Scheme 2). The structural-property relationships and gas separation behaviors of newly developed PI-ionenes and composites containing ILs are analyzed.

## 2. Materials and Methods

### 2.1. Materials

2-fluoronitrobenzene (“2-FNB”) (99%), 3-fluoronitrobenzene (“3-FNB”) (99%), and 4-fluoronitrobenzene (“4-FNB”) (98%) were purchased from Oakwood Chemical (Estill, SC, USA). Imidazole (99%) was purchased from Aldrich (St. Louis, MO, USA). Potassium carbonate (99%, anhydrous) and Pd/C (10% on C, Type 487) were purchased from BeanTown Chemical (Hudson, NH, USA). α,α’-Dichloro-p-xylene. (pDCXy, >98%), α,α’-Dichloro-m-xylene. (mDCXy, >96%), and α,α’-Dichloro-o-xylene (oDCXy, >97%) were purchased from TCI (Tokyo Chemical Industry) (Portland, OR, USA). Lithium bis-trifluoromethanesulfonimide (HQ-115) was purchased from 3M (Minneapolis, MN, USA). Ethanol (EtOH, 200 proof), *N*-methylpyrrolidone (NMP, ACS grade), dimethyl sulfoxide (DMSO, ACS grade), diethyl ether (Et_2_O) and toluene (anhydrous) were purchased from VWR (Atlanta, GA, USA). Acetic anhydride (Ac_2_O, >99%) was purchased from Alfa Aesar (Tewksbury, MA, USA). SiO_2_ (Celite 545) was purchased from Acros Organics. (Geel, Belgium) 4,4′-(hexafluoroisopropylidene)diphthalic anhydride (6FDA) was purchased from Akron Polymer Systems (Akron, OH, USA). All materials were used as obtained, without further purification.

### 2.2. Characterization

^1^H-NMR and ^13^C-NMR data were obtained using 360 MHz or 500 MHz Bruker Avance instruments. FT-IR data were collected on a Perkin Elmer Spectrum Two ATR FT-IR (Shelton, CT, USA). The thermal stabilities of these PI-ionenes with ILs were evaluated by thermogravimetric analysis (TGA) at a heating rate of 10 °C min^−1^ under an N_2_ atmosphere (Seiko TG/DTA 7300). The glass transition temperature (T_g_) of each PI-ionene was observed by DSC (TA Instruments, DSC Q20) from 20 to 300 °C with a scan rate of 10 °C min^−1^ under N_2_. The wide-angle X-ray diffraction (WAXD) patterns of the materials were measured using a Bruker D8 Discover diffractometer by employing a scanning rate of 4° min^−1^ in a 2*θ* range from 5° to 70° with a Co Kα1 X-ray (λ = 0.17886 nm) source. The d-spacing values were calculated using Bragg’s law (d = λ/2sin *θ*) and the Diffrac-EVA software. The number average molecular weight (M_N_) values of the PI-ionenes were determined via MALDI-TOF MS (Bruker Ultraflex). 

### 2.3. Synthesis of Imidazole-Aniline Precursors

#### 2.3.1. Synthesis of 4-(1H-imidazol-1-yl)aniline “I4A”

The synthesis of 4-(1H-imidazol-1-yl)aniline (I4A) starting from imidazole and 4-FNB was reported in our previous work [35]. ^1^H NMR (500 MHz, DMSO-*d*_6_) *δ* 7.95 (s, 1H), 7.48 (s, 1H), 7.22 (d, 2H), 7.01 (s, 1H), 6.64 (d, *J* = 8.8, 2H), 5.26 (s, 2H).

#### 2.3.2. Synthesis of 3-(1H-imidazol-1-yl)aniline “I3A”

The meta-derivative (I3A) was synthesized by a similar method. Imidazole (48.25 g, 709 mmol), 3-FNB (50 g, 354 mmol), and K_2_CO_3_ (53.76 g, 389 mmol) were added with 350 mL of DMSO to a 1000 mL round-bottom-flask. The vessel was capped with a rubber stopper and vented with a needle through the cap, to prevent the buildup of pressure in the flask upon heating. The reaction was heated to 110 °C overnight. The reaction was cooled to room temperature and poured into 600 mL of DI H_2_O to precipitate the product and remove excess imidazole. The product was filtered and then stirred in 250 mL of Et_2_O for 24 h to remove unreacted 3-FNB. The purified and dried product was collected as bright yellow powder. The imidazole-nitrophenyl product was reduced in EtOH (350 mL) with Pd/C (1.70 g), with a H_2_ feed (30 psi). The product was recovered and purified as a tan solid (49.9 g, 89%). ^1^H NMR (360 MHz, DMSO-*d*_6_) *δ* 8.08 (s, 1H), 7.57 (t, *J* = 1.3 Hz, 1H), 7.13 (t, *J* = 1.3 Hz, 1H), 7.08 (s, 2H), 6.73 (t, 1H), 6.72–6.67 (m, 1H), 6.60–6.53 (m, 1H), 5.41 (s, 2H).

#### 2.3.3. Synthesis of 2-(1H-imidazol-1-yl)aniline (“I2A”)

The synthesis of 2-(1H-imidazol-1-yl)aniline (I2A) starting from imidazole and 2-FNB was reported in our previous work [35]. ^1^H NMR (360 MHz, DMSO-*d*_6_) δ 7.74 (t, *J* = 1.1 Hz, 1H), 7.30 (t, *J* = 1.3 Hz, 1H), 7.14 (ddd, J = 8.1, 7.3, 1.6 Hz, 1H), 7.10 (t, J = 1.1 Hz, 1H), 7.03 (dd, J = 7.8, 1.6 Hz, 1H), 6.86 (dd, *J* = 8.1, 1.4 Hz, 1H), 6.64 (td, *J* = 7.5, 1.4 Hz, 1H), 5.06 (s, 2H).

### 2.4. Synthesis of Aromatic Polyimide Monomers

#### 2.4.1. Synthesis of 5,5′-(perfluoropropane-2,2-diyl)bis(2-(4-(1H-imidazol-1yl)phenyl)isoindoline-1,3-dione) (“6FDA-I4A”)

The synthesis of 5,5′-(perfluoropropane-2,2-diyl)bis(2-(4-(1H-imidazol-1yl)phenyl)isoindoline-1,3-dione) (“6FDA I4A”) from 6FDA and I4A was previously introduced in our previous work [33]. ^1^H NMR (360 MHz, DMSO-*d*_6_) δ 8.34 (d, 2H), 8.25 (d, 2H), 8.02 (d, 2H), 7.88–7.75 (m, 8H), 7.62 (dt, 2H), 7.17 (s, 4H). ^13^C NMR (126 MHz, DMSO-*d_6_*) δ 166.50, 166.35, 149.34, 136.94, 136.18, 135.73, 134.47, 133.63, 132.37, 130.66, 130.16, 129.28, 128.50, 118.74, 118.36, 113.03, 65.95.

#### 2.4.2. Synthesis of 5,5′-(perfluoropropane-2,2-diyl)bis(2-(3-(1H-imidazol-1-l)phenyl)isoindoline-1,3-dione) (“6FDA-I3A”)

5,5′-(perfluoropropane-2,2-diyl)bis(2-(3-(1H-imidazol-1-l)phenyl)isoindoline-1,3-dione) (“6FDA I3A”) was synthesized according to a similar procedure. 6FDA (13.95 g, 31.4 mmol) was added to a 250 mL round-bottom flask with 50 mL of DMF. The reaction was cooled to 5 °C and stirred for 1 h. I3A (10.00 g, 62.8 mmol) was then added to the flask, and the vessel was allowed to warm to room temperature overnight while stirring. Toluene (15 mL) was then added to the flask and the reaction was equipped with a reflux condenser. The vessel was then heated at 125 °C for 24 h. The solution was then cooled, and toluene and some DMF were removed via rotary evaporation. The remaining solution was poured into 600 mL of DI H_2_O to precipitate the product. The solids were filtered and washed with 2 × 100 mL of DI H_2_O, collected and dried for 24 h at 120 °C under vacuum, yielding the product as an off-white powder (13.9 g, 61%). ^1^H NMR (500 MHz, DMSO-*d*_6_) *δ* 8.29–8.22 (m, 4H), 8.03 (d, *J* = 8.0 Hz, 2H), 7.84–7.77 (m, 6H), 7.75–7.62 (m, 4H), 7.50–7.45 (m, 2H), 7.16 (s, 2H). ^13^C NMR (500 MHz, DMSO-*d*_6_) δ [ppm] 166.20, 152.99, 135.94, 133.38, 130.97, 130.24, 126.47, 125.08, 124.25, 120.87, 119.96, 119.96, 118.53, 116.08, 107.73, 97.76, 70.20. [M][H^+^]: calculated = 726.1450; found = 726.1439.

#### 2.4.3. Synthesis of 5,5′-(perfluoropropane-2,2-diyl)bis(2-(2-(1H-imidazol-1yl)phenyl)isoindoline-1,3-dione) (“6FDA I2A”)

5,5′-(perfluoropropane-2,2-diyl)bis(2-(2-(1H-imidazol-1yl)phenyl)isoindoline-1,3-dione) (“6FDA I2A”) was synthesized according to a similar procedure. 6FDA (4.00 g, 9.00 mmol) was added to a round-bottom flask with 30 mL of DMF. The reaction was cooled to 5 °C and stirred for 1 h. I2A (3.01 g, 18.9 mmol) was then added to the flask, and the vessel was allowed to warm to room temperature and stirred overnight. Toluene (10 mL) was then added to the flask, and the reaction was equipped with a reflux condenser. The vessel was then heated at 125 °C for 24 h. The solution was cooled, and toluene and some DMF were removed via rotary evaporation. The remaining solution was poured into 400 mL of DI H_2_O to precipitate the product. The solids were filtered and washed with 2 × 100 mL of DI H_2_O, collected, and dried for 24 h at 120 °C under vacuum. The solids were added to a round-bottom flask with a reflux condenser and 25 mL of Ac_2_O. To complete ring closure from the amic acid to the imide function, the solution was heated to 75 °C for 24 h. The product was precipitated in and washed with DI H_2_O, followed by drying at 120 °C overnight yielding the product as a crystalline brown powder (5.80 g, 88%). ^1^H NMR (500 MHz, DMSO-*d*_6_) δ 8.93–8.84 (m, 2H), 8.46 (m, 2H), 8.17–7.95 (m, 6H), 7.85–7.75 (m, 5H), 7.75–7.69 (m, 4H). ^13^C NMR (500 MHz, DMSO-*d*_6_) *δ* [ppm] 165.36, 158.72, 154.60, 134.53, 131.24, 130.34,129.77, 129.68, 129.49, 129.30, 127.31, 125.34, 124.20, 122.94, 120.08, 118.36, 116.41, 115.23, 71.55. [M][H^+^]: calculated = 726.1450; found = 726.1443.

### 2.5. Formation of Polyimide-Ionenes

#### 2.5.1. Synthesis of [6FDA I4A pXy][Tf_2_N]

6FDA-I4A ionenes were synthesized according to Scheme 2 using the following procedure: 6FDA-I4A (15.00 g, 20.6 mmol) and pDCXy (3.614 g, 20.6 mmol) were added with 180 mL of anhydrous DMF to a round-bottom heavy-walled pressure vessel and was sealed. The reaction was heated to 120 °C for 24 h. The product (as Cl^−^ salt) was precipitated in an Erlenmeyer flask containing 500 mL of DI water with 3 eq. of LiTf_2_N (14.82 g, 51.6 mmol). The [6FDA I4A pXy] ionene solution was stirred for 24 h to allow for the anion exchange. The product was then filtered and dried in a vacuum oven at 120 °C overnight. ^1^H NMR (500 MHz, DMSO-d_6_) δ 10.04 (br, 2H), 8.38 (br, 2H), 8.23 (br, 2H), 8.05 (br, 2H), 7.99–7.93 (m, 6H), 7.86–7.82 (br, 2H), 7.80–7.74 (br, 4H), 7.62 (br, 4H), 5.54 (br, 4H).

#### 2.5.2. Synthesis of [6FDA I3A mXy][Tf_2_N]

The meta- derivative was synthesized via a similar procedure. 6FDA-I3A (6.00 g, 8.26 mmol) and mDCXy (1.45 g, 8.26 mmol) were added with 80 mL of anhydrous DMF to a round-bottom heavy-walled pressure vessel and was sealed. The reaction was heated to 120 °C for 24 h. The product (as Cl^−^ salt) was precipitated in an Erlenmeyer flask containing 400 mL of DI water with 3 eq. of LiTf_2_N (7.12 g, 24.8 mmol). The [6FDA I3A mXy] ionene solution was stirred for 24 h to allow for the anion exchange. The product was then filtered and dried in a vacuum oven at 120 °C overnight. ^1^H NMR (500 MHz, DMSO-*d*_6_) *δ* 10.04 (s, 2H), 8.32 (s, 2H), 8.25 (d, *J* = 8.0 Hz, 2H), 8.05 (s, 2H), 8.03–7.90 (m, 6H), 7.90–7.83 (m, 4H), 7.73 (s, 1H), 7.71 (d, *J* = 7.3 Hz, 2H), 7.54 (m, 3H), 5.56 (s, 4H).

#### 2.5.3. Synthesis of [6FDA I2A oXy][Tf_2_N]

The ortho- derivative was synthesized via a similar procedure. 6FDA-I2A (6.00 g, 8.26 mmol) and oDCXy (1.45 g, 8.26 mmol) were added with 80 mL of anhydrous DMF to a round-bottom heavy-walled pressure vessel and was sealed. The reaction was heated to 120 °C for 24 h. The product (Cl¯ salt) was precipitated in an Erlenmeyer flask containing 400 mL of DI water with 3 eq. of LiTf_2_N (7.12 g, 24.8 mmol). The [6FDA I2A oXy] ionene solution was stirred for 24 h to allow for the anion exchange. The product was then filtered and dried in a vacuum oven at 120 °C overnight. ^1^H NMR (500 MHz, DMSO-*d*_6_) *δ* 9.59 (s, 2H), 8.87 (dd, *J* = 14.5, 6.9 Hz, 2H), 8.44 (m, 4H), 8.14–8.04 (m, 4H), 8.01 (m, 2H), 7.96 (s, 2H), 7.85–7.76 (m, 4H), 7.76–7.68 (m, 4H), 4.85 (s, 4H).

### 2.6. Preparation of Ionic Liquids

**Figure 1:**
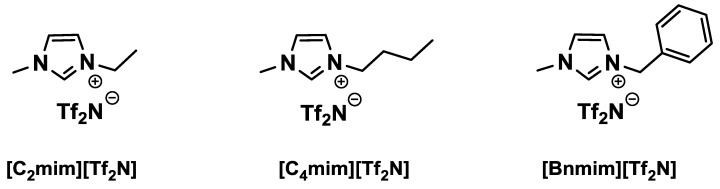
[Rmim][Tf_2_N] ionic liquids (ILs) utilized to form polyimide-ionene + IL composites.

The synthesis of 3-ethyl-1-methyl-1H-imidazol-3-ium bis((trifluoromethyl) sulfonyl)amide ([C_2_mim][Tf_2_N]), 3-butyl-1-methyl-1H-imidazol-3-ium bis((trifluoromethyl) sulfonyl)amide ([C_4_mim][Tf_2_N]), and 3-butyl-1-methyl-1H-imidazol-3-ium bis((trifluoromethyl) sulfonyl)amide ([Bnmim][Tf_2_N]) followed procedures previously introduced in the literature [24]. The ionic liquids shown in Figure 1 were incorporated as discussed in the following section on membrane formation.

### 2.7. Membrane Preparation

All the neat PI-ionenes and PI-ionene + IL membranes were prepared from solution-casting in *N,N*-dimethylacetamide (DMAc). As depicted in Table 1, the composite PI-ionene + IL membranes were prepared by combining two different equimolar ratios of the ILs with respective PI-ionenes. The membranes were fabricated as follows: the PI-ionenes were added to 15 mL centrifuge tubes, followed by the addition of the corresponding amount of an IL. DMAc was then added to bring the total volume to 9 mL for the composites and 7.5 mL for the neat dope solutions. The tubes were placed in a hot water bath for 2 h, to promote the dissolution and distribution of the solute. The tubes were then centrifuged for 5 min at 6000 rpm to separate any undissolved solids. The solution was then poured into (diameter = 60 mm) wells in a Teflon mold. The molds were then heated to 40 °C in a vacuum oven for 24 h. The temperature was slowly raised to 65, 85 °C, and then 110 °C over the course of 72 h in order to remove the solvent. The films were then peeled from the wells or collected into small discs of homogenized material. For uniformity, the membranes were then melt-pressed on a Carver press between sheets of Teflon, with the plate temperatures ranging from 75–90 °C under minimal pressure. The film thickness was controlled to be ~90–120 μm by adjusting the casting solution concentration.

### 2.8. Gas Separation Measurements

The gas permeation behaviors of newly developed PI-ionenes and PI-ionene + IL membranes were studied using a high-vacuum time lag apparatus based on the constant-volume/variable pressure method, as described in our previous work [34,35,36]. In this study, all measurements were ideal (i.e., single-gas) and performed at 20 °C, and the feed pressure was ~2 atm (~30 psia) against initial downstream vacuum (< 0.01 psia). Membranes were “masked” on both sides using an adhesive aluminum tape in order to confine gas permeation through a fixed membrane area either of 3/8” or 1/2” diameter [37]. Gases were tested in the order of N_2_, CH_4_, and CO_2_. The membrane sample was carefully evacuated before each cycle of gas permeation tests in order to remove any residual dissolved gas species. The permeability coefficient was determined from the linear slope of the downstream pressure rise versus the time plot (dp/d*t*) according to the following equation:

$$P=\frac{273}{76}\times \frac{Vl}{ATp_{o}}\times \frac{dp}{dt}$$

where *P* is the permeability expressed in Barrer (1 barrer = 10^−10^ (cm^3^_STP_ cm)/(cm^2^ s cmHg); *V* (cm^3^) is the downstream volume; *l* (cm) is the membrane thickness; *A* (cm^2^) is the effective area of the membrane; *T* (K) is the temperature of measurement; *p_o_* (Torr) is the pressure of the feed gas in the upstream chamber, and d*p*/d*t* is the rate of the pressure rise under steady state. For a given gas pair (e.g., i/j), the permselectivity (αi,j) was calculated as Pi/Pj from the pure-gas permeability.

## 3. Results and Discussion

### 3.1. Characterization

The purity, molecular structure, and thermophysical properties of both the neat PI-ionenes and composites with ILs were thoroughly investigated. ^1^H-NMR and ^13^C-NMR were utilized for structural confirmation of the monomers and polyimide-ionenes. NMR chemical shifts [δ] are reported in the experimental section, with spectra included in Figures S1–S6. ^1^H-NMR of the monomers supports formation of bis-imidazole imide monomers, indicated by the disappearance of the broad NH_2_ peak and a downfield shift of all peaks in the aromatic region as a result of imidization. Additionally, the chemical shifts between 9.59–10.04 ppm exhibited by the PI-ionenes are characteristic of imidazolium protons, indicating successful formation of imidazolium moieties upon polymerization via the Menshutkin reaction.

The structures of each PI-ionene, neat and with ILs, were further confirmed by FT-IR data (See Figure S7). All derivatives support the incorporation of the Tf_2_N^–^ anion (SO_2_ stretching vibrations at 1180 and 1050 cm^−1^, SNS stretching at 725 cm^−1^, CF_3_ stretching at 1370 cm^−1^), incorporated in the backbone of the neat polymers and the counter ion of all three imidazolium ionic liquids [33,38]. C–H stretching is observed, intensified by the incorporation of C_2_mim, C_4_mim, and Bnmim. Peaks corresponding to the imide functionality are observed by C-N stretching around 1350 cm^−1^ and C=O stretching at 1720 cm^−1^_._

### 3.2. Thermal Characterization

Differential scanning calorimetry (DSC) was utilized to determine T_g_ values for the three PI-ionenes and aforementioned IL hybrids. The thermal data are summarized in Table 2, with plots included in a supporting document (See Figure S8–S21). The *d*-spacing values for all derivatives are also summarized in Table 2, and discussed later in Section 3.3. The effects of the substitution pattern (i.e., *para-, meta-, ortho* connectivity) and IL content were studied. The [6FDA I4A pXy][Tf_2_N] polymer exhibited the lowest T_g_ of the neat derivatives, while the T_g_ of [6FDA I3A mXy][Tf_2_N] was the highest. Due to its symmetric nature, the *para*- derivatives had the lowest T_g_ values indicating better chain mobility. The inherent kinks of the *meta*- derivatives may promote better chain entanglement. The *ortho*- derivatives were all very brittle, even at elevated temperatures and with IL, and showed the highest T_g_ values. This may be attributed to poor chain entanglement and mobility due to the sterically strained linkages. Generally, the incorporation of 1 eq. of IL per polymer repeat unit lowered the T_g_, in comparison to the neat material. However, when comparing derivatives **2,3** and **4,5**, the T_g_ observed is higher in hybrids containing 2 eq. of IL versus 1 eq. The stoichiometric pairing of 2 eq. of imidazolium IL with each repeat unit (containing two imidazolium groups along the backbone) seems to stabilize the material due to interactions between the ionic groups serving as a non-covalent “crosslink”. There is no consistent trend correlating the effect of IL structure to T_g_, but with [6FDA I4A pXy][Tf_2_N] +IL hybrids increasing T_g_ corresponds to C_4_mim < C_2_mim < Bnmim. The aliphatic portion of C_2_mim and C_4_mim may allow for greater mobility between or amongst chains than the aromatic counterpart Bnmim. These glass transitions occur in comparable temperature ranges as partially aromatic PIs and similar ionic materials, yet the T_g_ values of these PI-ionene composites are higher than many ionic liquid-based materials due to the incorporation of the imide linkages and aromatic content [3,15,32,39,40,41]. In comparison to rigid, 6FDA-based polyimide materials with no ionic content, the T_g_ values are markedly lower, which may aid in processability [11,14,42,43].

**Figure 2:**
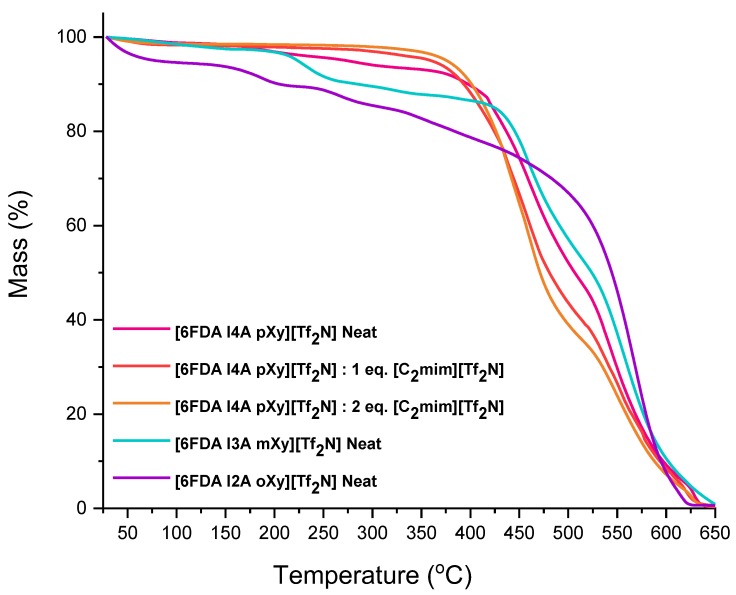
thermogravimetric analysis (TGA) data for polyimide-ionene derivatives, neat or with specified IL content.

The effects of backbone connectivity and interactions with ILs can also be seen in the TGA data shown in Figure 2. [6FDA I2A oXy][Tf_2_N] (11) exhibited poor thermal stability overall, with the earliest onset of degradation. Some of the early mass lost is most likely due to trapped H_2_O and DMF; however, the steric strain and poor chain entanglement of the ortho-substitution propagating along the ionene backbone results in gradual degradation at elevated temperatures (T_d, 10%_ = 204 °C, T_d, 25%_ = 443 °C). [6FDA I3A mXy][Tf_2_N] (**7**) showed improved stability, with a small loss from solvent and polymer mass around 217 °C followed by a more significant onset of degradation around 416 °C (T_d, 10%_ = 283 °C, T_d, 25%_ = 456 °C). The highest onset of degradation was observed in the [6FDA I4A mXy][Tf_2_N] derivatives, even with IL present in the composite material. Neat [6FDA I4A pXy][Tf_2_N] (**1**) was stable until 415 °C, followed by a steady decline in mass (T_d, 10%_ = 283 °C, T_d, 25%_ = 456 °C). TGA data supported the integration of IL into the PI-ionenes, with 1–2 eq. of C_2_mim with [6FDA I4A pXy][Tf_2_N] (**2**,**3**) stabilizing the material up to 375 °C. The stoichiometric pairing of 2 eq. of IL versus 1 eq. with the same backbone results in the onset of degradation to slightly higher temperatures. Thus, the [6FDA I3A mXy][Tf_2_N] ionenes are comparably stable with 1 eq. C_2_mim (T_d, 10%_ = 393 °C, T_d, 25%_ = 435 °C) or 2 eq. C_2_mim (T_d, 10%_ = 400 °C, T_d, 25%_ = 434 °C). The inflection point observed upon 50% mass loss is indicative of the breakdown of the Tf_2_N anion (contributes ~ 60 wt % per repeat unit) initially, coinciding with the rapid decomposition of the backbone that follows. These materials exhibit excellent thermal stability for ionic polymers, with thermal decomposition behavior comparable to primarily aromatic polyimides [3]. The connectivity through the N atoms of the imidazolium segment and the CH_2_ groups incorporated within the xylyl linkage lower the stability of these materials if compared to wholly aromatic, rigid PIs.

### 3.3. Structural Characterization

**Figure 3:**
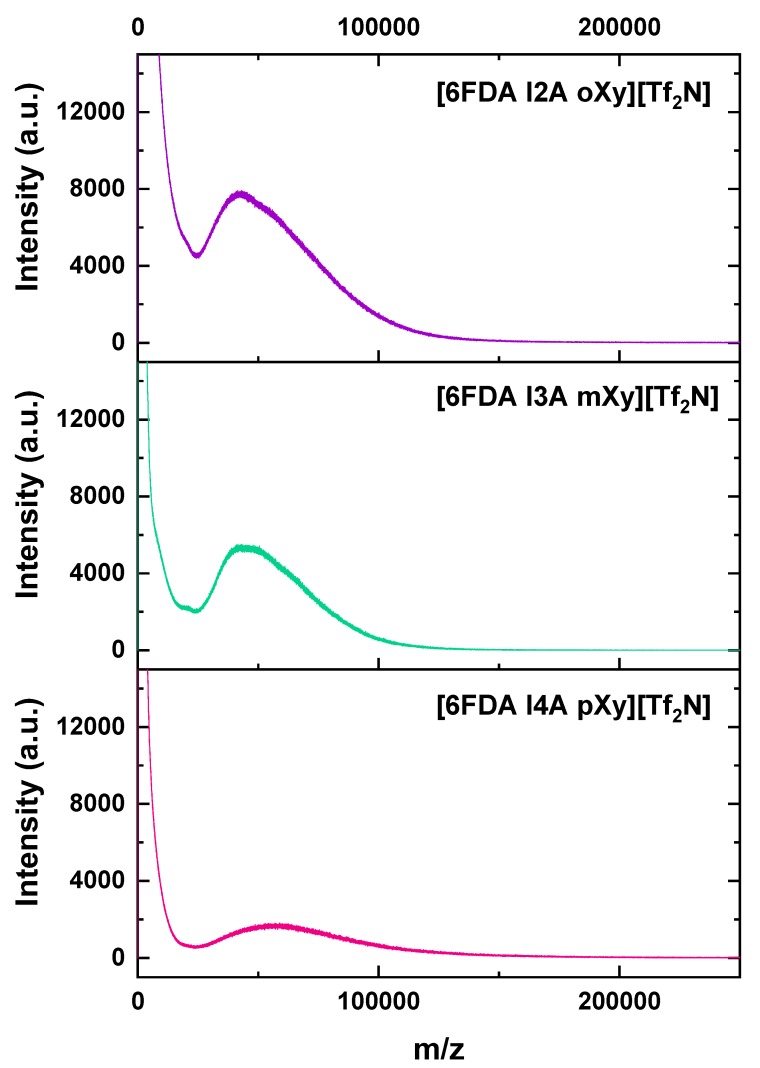
MALDI-TOF MS spectra for PI-ionenes.

M_N_ values of the three, neat polyimide-ionenes were determined using MALDI-TOF MS. The M_N_ values obtained from the plots included in Figure 3 ranged from 44–59 kDa, which corresponds to the number average degree of polymerization values (X_N_) ranging from 30 to 45 repeat units. [6FDA I2A oXy][Tf_2_N] showed the lowest M_N_ (~44 kDa)_,_ with a significant portion of smaller oligomers due to the steric hinderance the *ortho*-substituted end groups in the monomer, dihalide linkage, and resultant polymer. The [6FDA I3A mXy][Tf_2_N] and [6FDA I4A pXy][Tf_2_N] materials proved to have higher molecular weights, with M_N_ values of ~46 kDa and ~59 kDa, respectively. The presence of oligomeric and low molecular weight content in the *meta*- and *ortho*- derivatives may be caused by decreased structural stability promoting fragmentation upon ionization.

**Figure 4:**
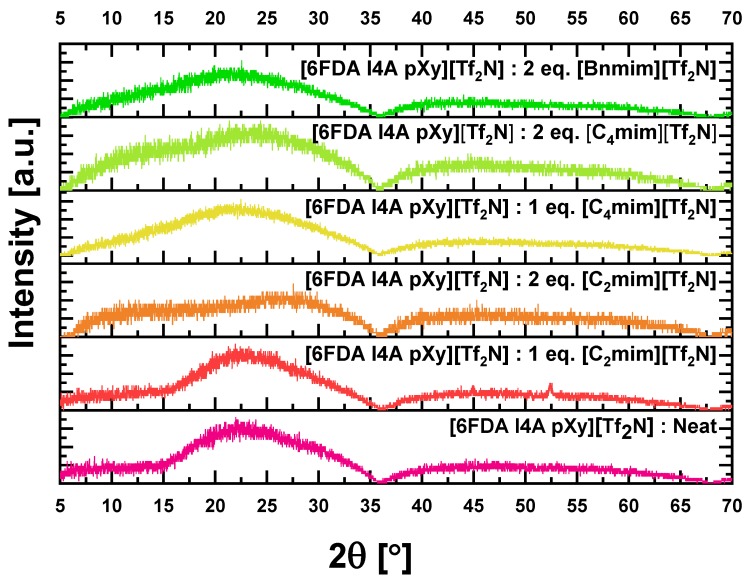
XRD profiles of the six [6FDA I4A pXy][Tf_2_N] derivatives.

X-ray diffraction (XRD) data were collected for all PI-ionene derivatives, to analyze the effects of backbone connectivity (i.e., *para, meta, ortho-)* and IL content on *d*-spacing. It has been shown that *d*-spacing can be correlated to interchain spacing in a polymeric network; thus, these trends indicate differences in the packing efficiency and are utilized here to predict which films would be expected to exhibit the highest permeability [44]. Figure 4 shows XRD spectra for the six [6FDA I4A pXy][Tf_2_N] derivatives for comparison. The other materials were also tested, with *d*-spacing values included earlier in Table 2 (See Figure S23). Each showed similar broad halos, with the peak of the main halo appearing between 2*θ* = 22°–27° and a secondary low-intensity halo spanning 2*θ* = 36°–67°. Bragg’s law was used to determine *d*-spacing values, utilizing the Diffrac.Eva software for accuracy. Due to the similarity in the backbone structure, the *d*-spacing values fall within a small range of 3.92–4.83 Å for the main halo. In most cases, the *d*-spacing decreases slightly with the incorporation of IL, as these free imidazolium cations migrate and fill the space between the ionene chains. As seen in **2–5**, the coordination between the backbone and the ILs is stronger with the pairing of 2 eq. of IL, with lower *d*-spacing values indicating that the chains are drawn closer around these ionic groups. The main halo is notably broadened (somewhat bimodal) by the incorporation of 2 eq. of IL, and the composite material is more rubbery or amorphous than the glassy neat polymers. The [6FDA I4A pXy][Tf_2_N] materials **1–6** exhibit the highest *d*-spacing values, though comparable to the [6FDA I2A oXy][Tf_2_N] materials **11–14.**

These structure-property characterizations were evaluated in order to understand the suitability of these PI-ionene + IL hybrids as gas separation membranes. Although [6FDA I3A mXy][Tf_2_N] and [6FDA I2A oXy][Tf_2_N] ionenes and their associated composites with ILs showed good thermal resistance and moderate stability, the lower molecular weight caused the processed films to be less mechanically stable and not adequate for gas separation performance. Thus, only the [6FDA I4A pXy][Tf_2_N] backbone was selected as the basis for comparison of permeability and selectivity in this newly developed PI-ionene series. It also should be mentioned that the stoichiometric pairing of 2 eq. of imidazolium ILs with each repeating unit of PI-ionene seemed more stable and tolerable as evidenced by DSC analysis. Therefore, [6FDA I4A pXy][Tf_2_N] ionene + IL composites having 2 eq. of imidazolium ILs were considered for further gas separation studies.

### 3.4. Gas Separation Performance

**Figure 5:**
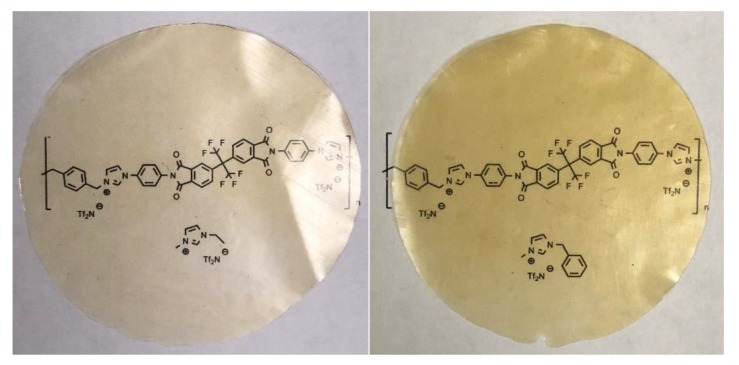
Membranes (~3′ in diameter) over their respective structures. [6FDA I4A pXy][Tf_2_N]: [C_2_mim][Tf_2_N] (**left**) and [6FDA I4A pXy][Tf_2_N]: [Bnmim][Tf_2_N] (**right**).

Images of full films of two [6FDA I4A pXy][Tf_2_N] ionene + IL composites with 2 eq. of imidazolium ILs are included in Figure 5.

The pure gas permeabilities and permselectivities of the [6FDA I4A pXy][Tf_2_N] ionene and IL-containing composites with [C_2_mim][Tf_2_N], [C_4_mim][Tf_2_N] and [Bnmim][Tf_2_N] were investigated using high-vacuum time-lag units at 3 atm and 20 °C (Table 3). However, the permeation results of [6FDA I4A pXy][Tf_2_N] composite membrane with [C_2_mim][Tf_2_N] is not presented in Table 3 because of inconsistency in the measured values. The [6FDA I4A pXy][Tf_2_N] with 2 eq. of [C_2_mim][Tf_2_N] was unstable under higher pressure, plausibly due to the extremely higher interchain packing (lowest *d*-spacing value of 3.92 Å was obtained for this combination, Table 2). Nonetheless, the other two hybrid-ionene membranes containing C_4_mim and Bnmim ILs exhibited significant changes in the separation properties of CO_2_ relative to N_2_ and CH_4_. The high CO_2_/CH_4_ selectivities are comparable to values reported for leading gas separation membranes; however, a significant increase in the permeability (10^2^–10^4^ barrer) would be required to compete with membranes at or over the Robeson Upper Bound [45].

As shown in Table 3, both the [C_4_mim][Tf_2_N] and [Bnmim][Tf_2_N] composites of [6FDA I4A pXy][Tf_2_N] displayed a drastic increase in the permeability of the CO_2_ compared to the neat [6FDA I4A pXy][Tf_2_N] membrane, probably due to the increased quadrupole interactions of ILs with CO_2_, as well as the versatility of the polymer matrix. The [C_4_mim][Tf_2_N] composite membrane obtained a ~2× increase (4.57 barrer) and the [Bnmim][Tf_2_N] composite displayed nearly a 3× increase (6.32 barrer) in their CO_2_ permeabilities. Unlike the high CO_2_ separation trends, the ability of the [6FDA I4A pXy][Tf_2_N] + IL composites to separate other nonpolar gases (N_2_ and CH_4_), appeared to be normal, and these materials yielded enhanced CO_2_ permeability without sacrificing their CO_2_/N_2_ and CO_2_/CH_4_ permselectivities (Table 3). Overall, the CO_2_ separation behaviors of both the C_4_mim and Bnmim containing hybrid membranes of newly developed [6FDA I4A pXy][Tf_2_N] ionene exhibited potential usefulness for CO_2_/light gas separation in the context of an appropriate structural and stoichiometric design.

## 4. Conclusions

In conclusion, a series of three 6FDA-based PI-ionenes and composites with ILs have been successfully synthesized, and structure-property relationships of substitution patterns with *para-, meta-*, *and ortho* connectivity, as well as the utility of the corresponding membranes for CO_2_ gas separation as neat ionene and IL hybrids forms were studied. The results showed that the newly developed 6FDA-based polyimide-ionenes were compatible with the ILs such as [C_2_mim][Tf_2_N], [C_4_mim][Tf_2_N] and [Bnmim][Tf_2_N], producing homogeneous hybrid membranes with the high thermal stability of ~380 °C. Specifically, [6FDA I4A pXy][Tf_2_N] ionene + IL composites showed promising performance for membrane-based CO_2_ separation with increased permeability of 2× and 3× for the [C_4_mim][Tf_2_N] and [Bnmim][Tf_2_N] composites, respectively, compared to the neat [6FDA I4A pXy][Tf_2_N]. The feasibility of using 6FDA-based PI-ionenes in combination with stable films composed of various ILs, which display a high affinity toward CO_2_, may enable the development of materials with a range of properties. Furthermore, in view of the versatility and functionality of the imidazolium platform, together with the ability to tune the structural-property relationships, PI-ionenes can be further developed for use in gas separation membranes.

## Figures and Tables

**Table 1:** Mass/Ratio calculations for the membrane fabrications.

Materials			Mass/Ratios		
ID	Polyimide-Ionene	IL (Equivalents)	Polyimide-Ionene [g]	IL [g]	V_total_ w/Solvent [mL]
**1**	[6FDA I4A pXy][Tf_2_N]	Neat	0.75	-	7.5
**2**		[C_2_mim][Tf_2_N] (1)	0.50	0.141	9
**3**		[C_2_mim][Tf_2_N] (2)	0.50	0.281	9
**4**		[C_4_mim][Tf_2_N] (1)	0.50	0.151	9
**5**		[C_4_mim][Tf_2_N] (2)	0.50	0.301	9
**6**		[Bnmim][Tf_2_N] (2)	0.50	0.326	9
**7**	[6FDA I3A mXy][Tf_2_N]	Neat	0.75	-	7.5
**8**		[C_2_mim][Tf_2_N] (2)	0.50	0.281	9
**9**		[C_4_mim][Tf_2_N] (2)	0.50	0.301	9
**10**		[Bnmim][Tf_2_N] (2)	0.50	0.326	9
**11**	[6FDA I2A oXy][Tf_2_N]	Neat	0.75	-	7.5
**12**		[C_2_mim][Tf_2_N] (2)	0.50	0.281	9
**13**		[C_4_mim][Tf_2_N] (2)	0.50	0.301	9
**14**		[Bnmim][Tf_2_N] (2)	0.50	0.326	9

**Table 2:** Glass transition and *d*-spacing values that characterize 6FDA polyimide-ionenes and their composites with IL.

ID	Polyimide-Ionene	IL (Equivalents)	Tg [°C]	*d*-spacing [Å]
**1**	[6FDA I4A pXy][Tf_2_N]	Neat	205.9	4.61
**2**		[C_2_mim][Tf_2_N] (1)	199.4	4.55
**3**		[C_2_mim][Tf_2_N] (2)	209.9	3.92
**4**		[C_4_mim][Tf_2_N] (1)	182.4	4.83
**5**		[C_4_mim][Tf_2_N] (2)	185.2	4.33
**6**		[Bnmim][Tf_2_N] (2)	218.9	4.73
**7**	[6FDA I3A mXy][Tf_2_N]	Neat	236.2	4.66
**8**		[C_2_mim][Tf_2_N] (2)	219.7	4.12
**9**		[C_4_mim][Tf_2_N] (2)	199.2	4.24
**10**		[Bnmim][Tf_2_N] (2)	185.5	4.11
**11**	[6FDA I2A oXy][Tf_2_N]	Neat	221.8	4.61
**12**		[C_2_mim][Tf_2_N] (2)	221.4	4.55
**13**		[C_4_mim][Tf_2_N] (2)	243.3	4.35
**14**		[Bnmim][Tf_2_N] (2)	236.3	4.26

**Table 3:** Pure Gas Permeabilities (P)^a^ and Permselectivities (α) for the neat [6FDA I4A pXy][Tf_2_N] ionene and composite membranes with [C_4_mim][Tf_2_N] and [Bnmim][Tf_2_N] at 3 atm and 20 °C ^b^.

[6FDA I4A pXy][Tf_2_N]	*P*_CO2_	*P*_N2_	*P*_CH4_	*α*_CO2/N2_	*α*_CO2/CH4_
**Neat**	2.15 ± 0.16	0.103 ± 0.01	0.161 ± 0.02	20.9 ± 2	13.4 ± 1
**[C_4_mim][Tf_2_N] (2 equiv.)**	4.57 ± 0.30	0.239 ± 0.02	0.189 ± 0.02	19.1 ± 1	24.8 ± 2
**[Bnmim][Tf_2_N] (2 equiv.)**	6.32 ± 0.15	0.282 ± 0.02	0.294 ± 0.02	22.4 ± 1	21.5 ± 1

^a^*P* in barrers, where 1 barrer = 10^−10^ (cm^3^_STP_ cm)/(cm^2^ s cmHg).

^b^Error represents one standard deviation.

## Supplementary Materials

The following are available online at https://www.mdpi.com/2077-0375/9/7/79/s1, Figure S1: ^1^H-NMR of I3A, S2: ^1^H-NMR of 6FDA I3A monomer, S3: ^1^H-NMR of 6FDA I2A monomer, S4: ^1^H-NMR of [6FDA I4A pXy][Tf_2_N] polyimide-ionene, S5: ^1^H-NMR of [6FDA I3A mXy][Tf_2_N] polyimide-ionene, S6: ^1^H-NMR of [6FDA I2A oXy][Tf_2_N] polyimide-ionene, S7: Compilation of IR spectra, S8: DSC plots for [6FDA I4A pXy][Tf_2_N] Neat, S9: DSC plots for [6FDA I4A pXy][Tf_2_N]: 1 eq. [C_2_mim][Tf_2_N], S10: DSC plots for [6FDA I4A pXy][Tf_2_N]: 2 eq. [C_2_mim][Tf_2_N], S11: DSC plots for [6FDA I4A pXy][Tf_2_N]: 1 eq. [C_4_mim][Tf_2_N], S12: DSC plots for [6FDA I4A pXy][Tf_2_N]: 2 eq. [C_4_mim][Tf_2_N], S13: S13: DSC plots for [6FDA I4A pXy][Tf_2_N]: 2 eq. [Bnmim][Tf_2_N], S14: DSC plots for [6FDA I3A mXy][Tf_2_N] Neat, S15: DSC plots for [6FDA I3A mXy][Tf_2_N]: 2 eq. [C_2_mim][Tf_2_N], S16: DSC plots for [6FDA I3A mXy][Tf_2_N]: 2 eq. [C_4_mim][Tf_2_N], S17: DSC plots for [6FDA I3A mXy][Tf_2_N]: 2 eq. [Bnmim][Tf_2_N], S18: DSC plots for [6FDA I2A oXy][Tf_2_N] Neat, S19: DSC plots for [6FDA I2A oXy][Tf_2_N]: 2 eq. [C_2_mim][Tf_2_N], S20: DSC plots for [6FDA I2A oXy][Tf_2_N]: 2 eq. [C_4_mim][Tf_2_N], S21: DSC plots for [6FDA I2A oXy][Tf_2_N]: 2 eq. [Bnmim][Tf_2_N], S22: MALDI-TOF spectra for the three, neat polyimide-ionenes, S23: XRD spectra for all samples, with 2θ values from 5-70°, S24: SEM image of [6FDA I4A pXy][Tf_2_N] (Neat), S25: SEM image of [6FDA I4A pXy][Tf_2_N]: [C_2_mim][Tf_2_N] (1 equivalent), S26: SEM image of [6FDA I4A pXy][Tf_2_N]: [C_2_mim][Tf_2_N] (2 equivalents), S27: SEM image of [6FDA I4A pXy][Tf_2_N]: [C_4_mim][Tf_2_N] (1 equivalent).
