# Supplementary material for: Synthesis and Performance of 6FDA-Based Polyimide-Ionenes and Composites with Ionic Liquids as Gas Separation Membranes
Source: Membranes (Basel). 2019 Jul 3;9(7):79. doi: 10.3390/membranes9070079 (PMC6681123; doi:10.3390/membranes9070079)
Supplement: Supplementary file 1 [file membranes-09-00079-s001.pdf]

SUPPORTING INFORMATION FOR

Synthesis and Performance of 6FDA-based Polyimide-Ionenes and Composites with Ionic Liquids as Gas Separation Membranes

Kathryn E. O'Harra,[a,1] Irshad Kammakakam,[a,1] Emily M. DeVriese,[a] Danielle M. Noll,[a] Jason E. Bara,[a*] and Enrique M. Jackson[b]

[a] *University of Alabama, Department of Chemical & Biological Engineering, Tuscaloosa, AL 35487-0203 USA*

[b] *NASA Marshall Space Flight Center, Huntsville, AL 35812 USA*

[*]Corresponding author's e-mail address: jbara@eng.ua.edu

[1]These authors contributed equally

Included below is relevant supporting data referenced in the manuscript: "Synthesis and Performance of 6FDA-based Polyimide-Ionenes and Composites with Ionic Liquids as Gas Separation Membranes". NMR, FT-IR, MALDI-TOF MS, DSC, XRD, and SEM results are shown in the following sections.

NMR Characterization

The ^{1}H-NMR and ^{13}C-NMR spectra for the monomers and polymers outlined in the manuscript are included below.

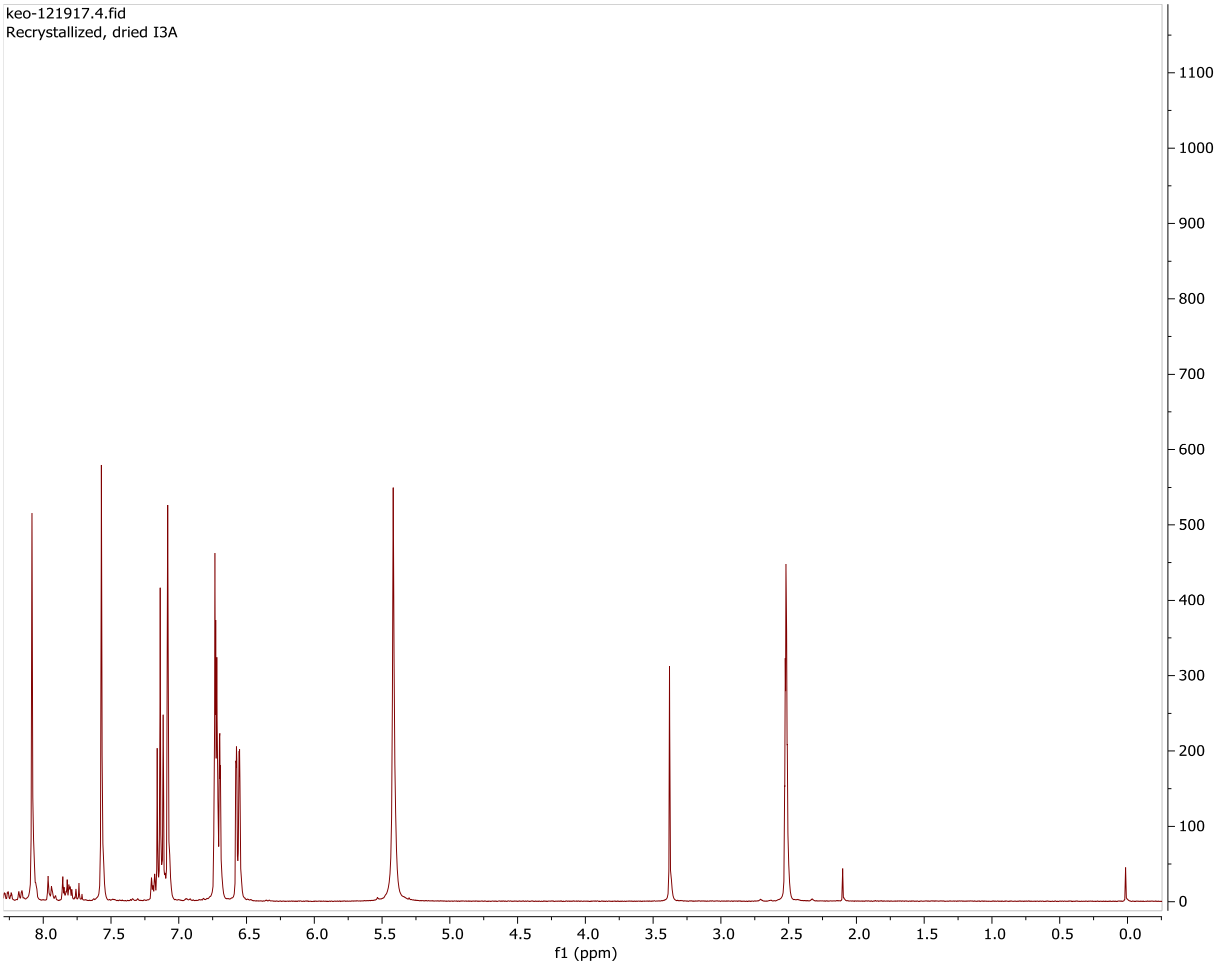

Figure S1: ^{1}H-NMR of I3A.

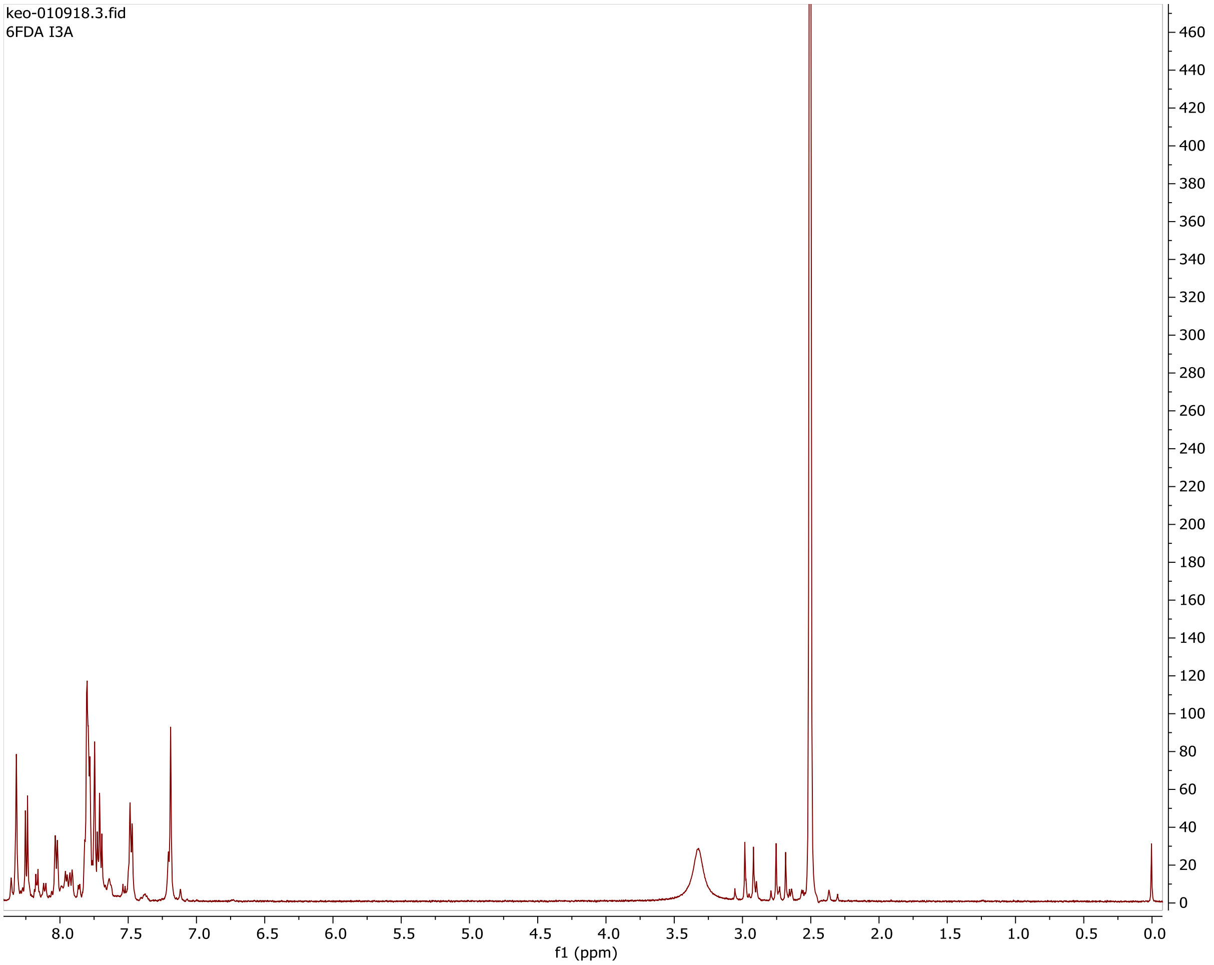

Figure S2: ^{1}H-NMR of 6FDA I3A monomer.

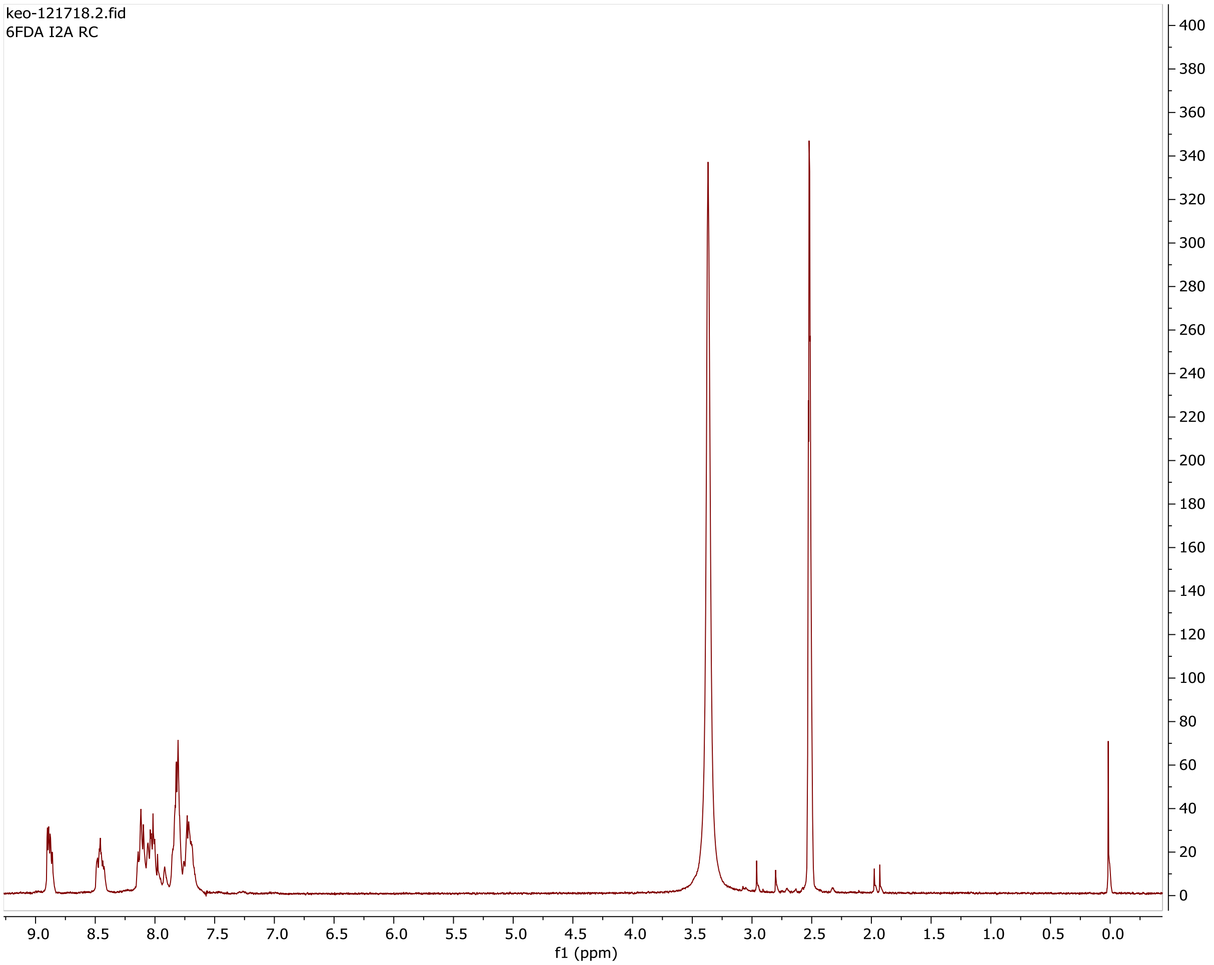

Figure S3: ^{1}H-NMR of 6FDA I2A monomer.

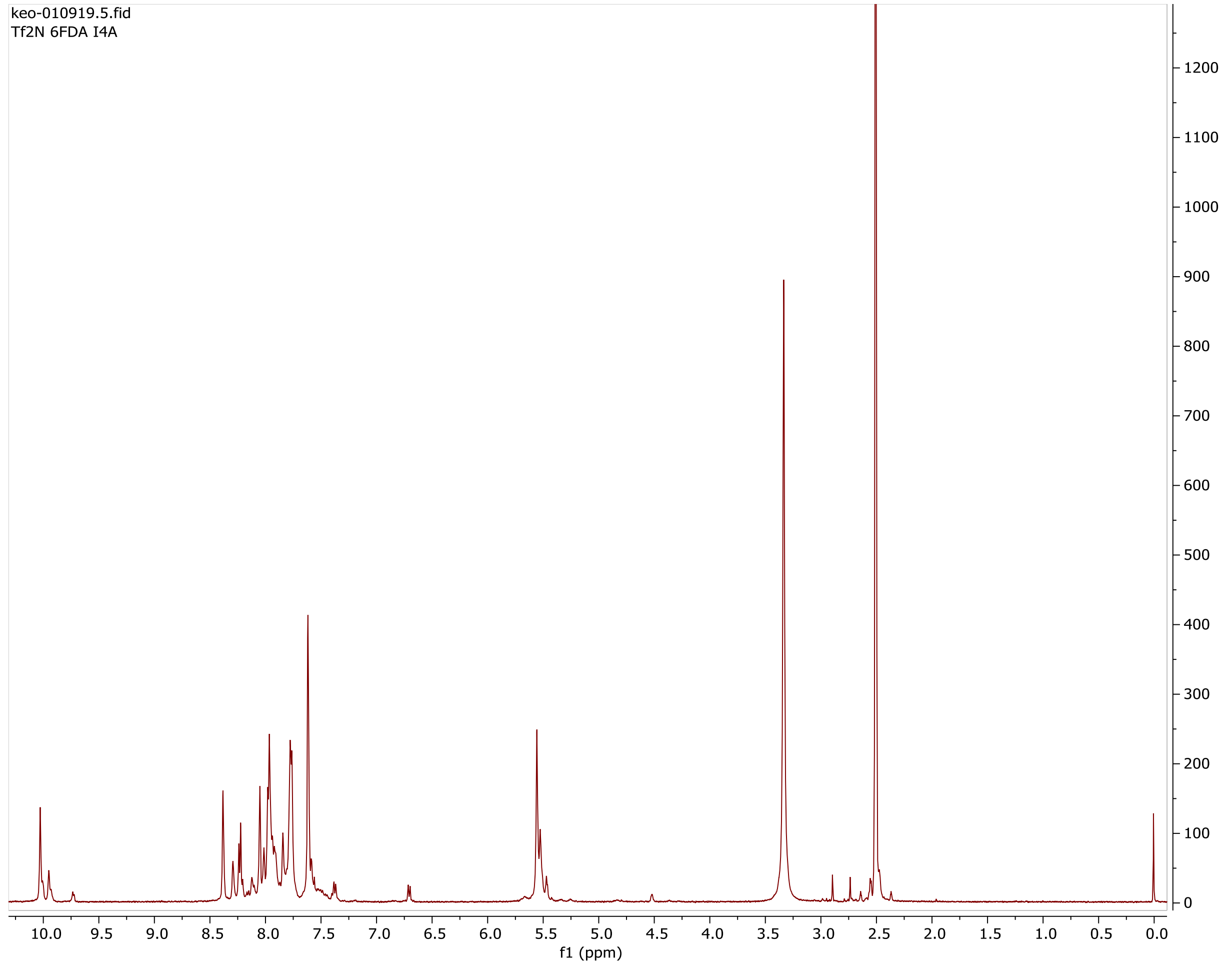

Figure S4: ^{1}H-NMR of [6FDA I4A pXy][Tf_2N] polyimide-ionene.

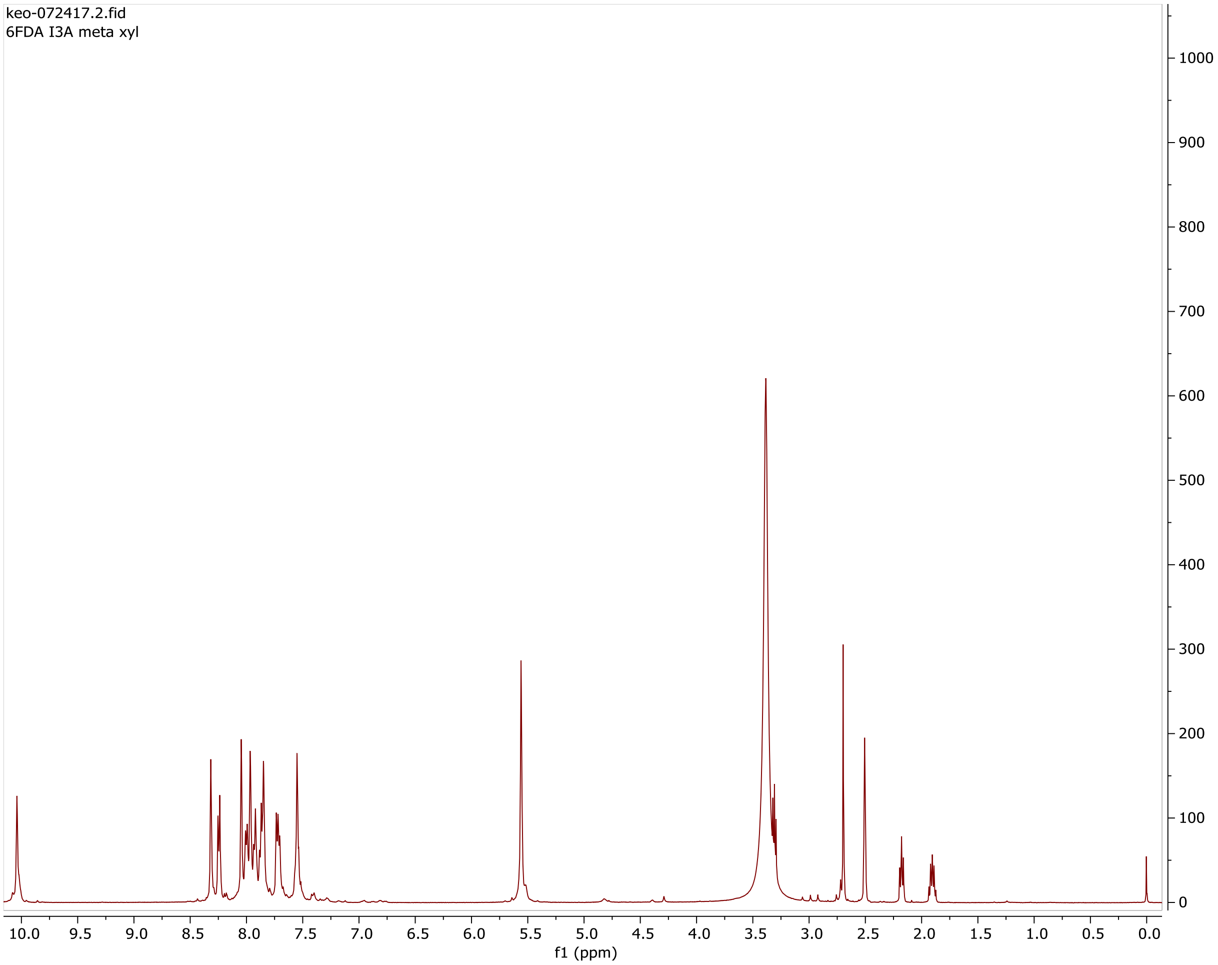

Figure S5: ^{1}H-NMR of [6FDA I3A mXy][Tf_2N] polyimide-ionene.

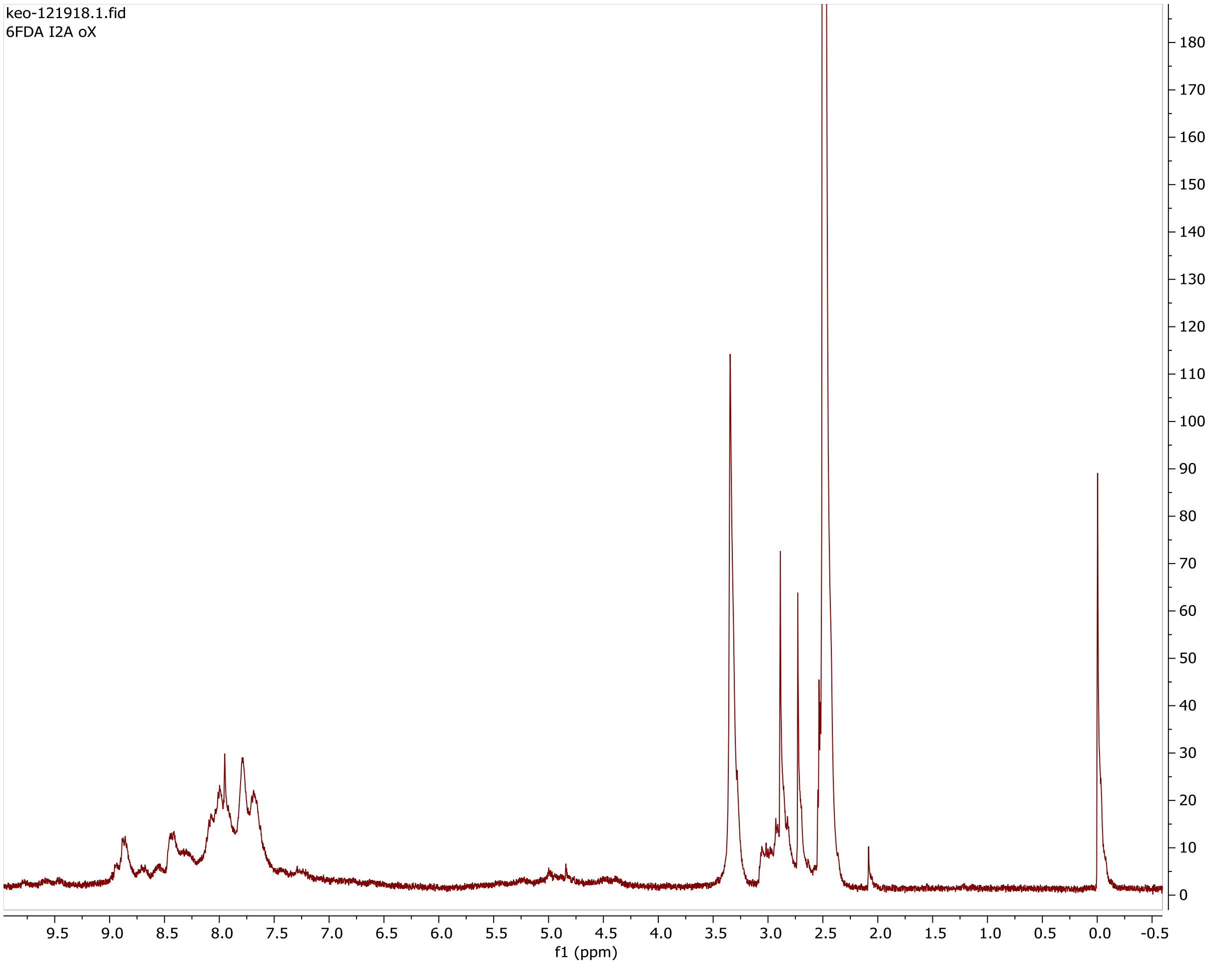

Figure S6: ^{1}H-NMR of [6FDA I2A oXy][Tf_2N] polyimide-ionene.

FT-IR Data

See Figure S7 for the FT-IR data for derivatives **1-14**. All samples were run on an ATR-FTIR.

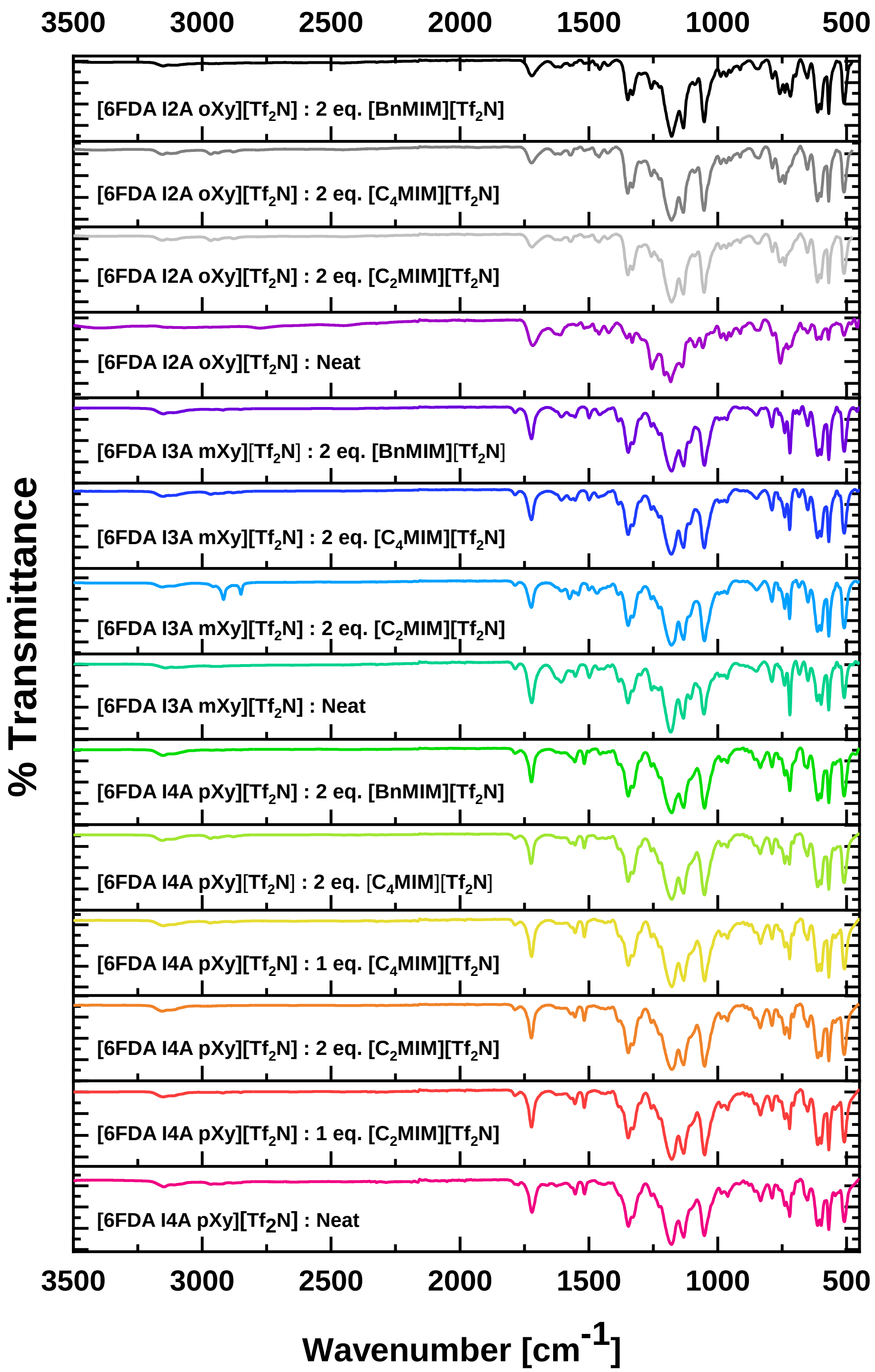

Figure S7: Compilation of IR spectra.

DSC Plots

The glass transition temperatures (T_g) were determined by DSC, as discussed in the manuscript. Each material was tested 2-3 times, for comparison and accuracy (Run 1: solid line; Run 2: dashed line; Run 3: dotted line). The temperature was increased from 0 – 300 °C, and all thermographs are plotted "exo up". In the first run, the scan was cycled from 0 °C to 300 °C, then back down to 25 °C raised back to 300 °C, to investigate the effects and consistency of data. Runs 2 and 3 used new material, and were only run from 0 °C to 300 °C for comparison of the observed transition temperatures. Figures S8-S21 show the various tests performed with portions of each sample.

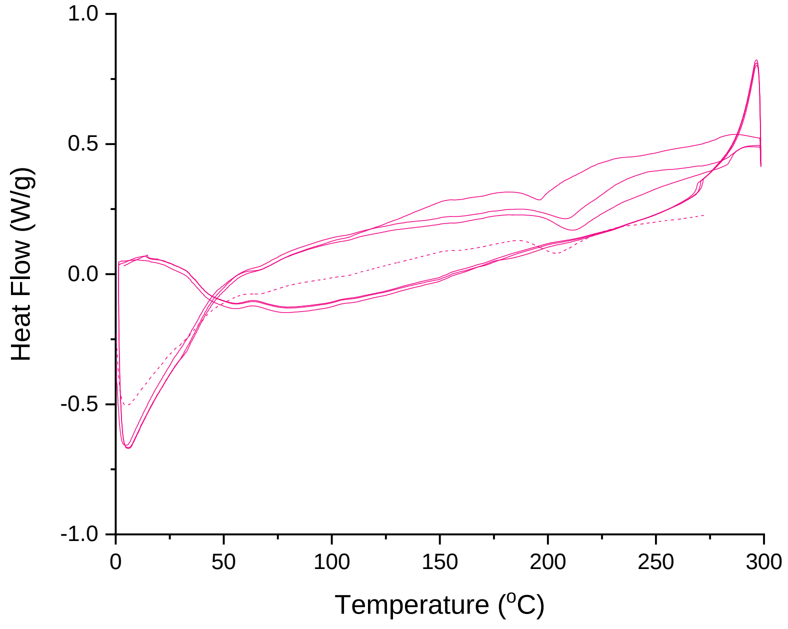

Figure S8: DSC plots for [6FDA I4A pXy][Tf_2N] Neat.

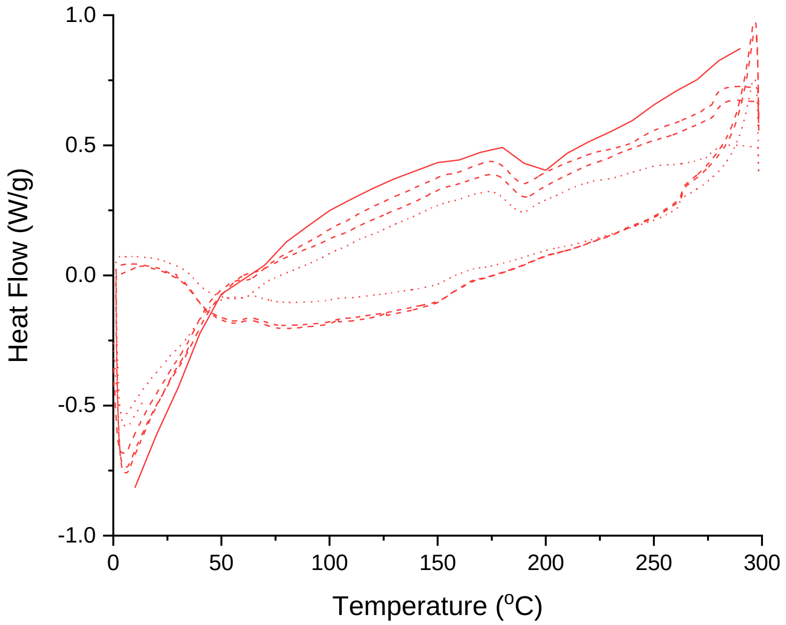

Figure S9: DSC plots for [6FDA I4A pXy][Tf_2N]: 1 eq. [C_2mim][Tf_2N].

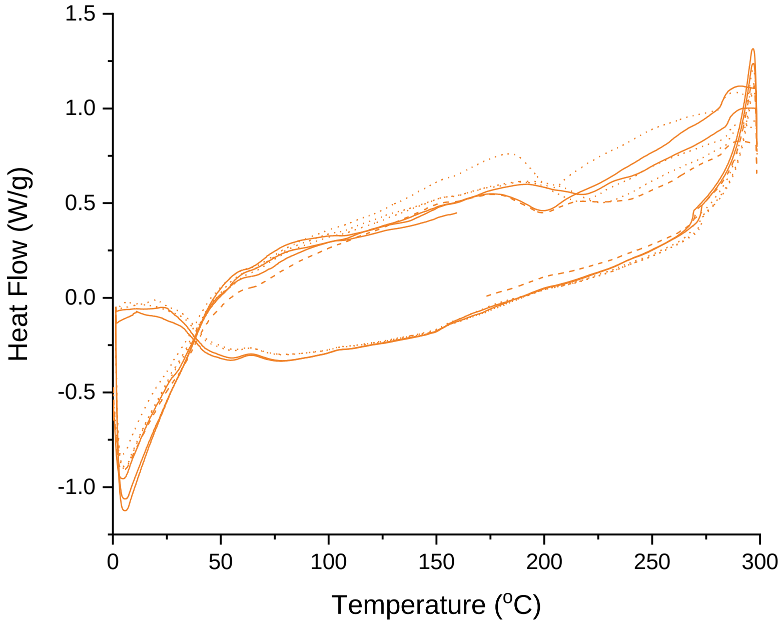

Figure S10: DSC plots for [6FDA I4A pXy][Tf_2N]: 2 eq. [C_2mim][Tf_2N].

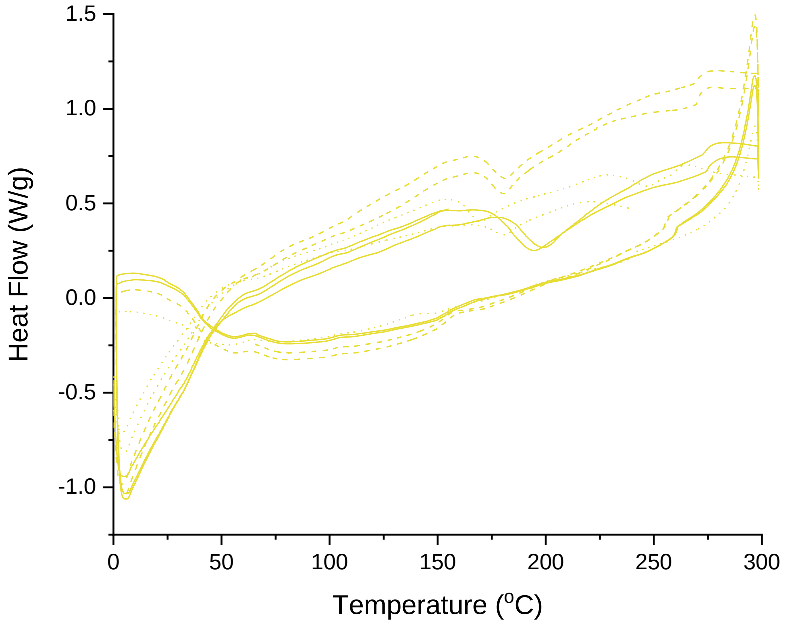

Figure S11: DSC plots for [6FDA I4A pXy][Tf_2N]: 1 eq. [C_4mim][Tf_2N].

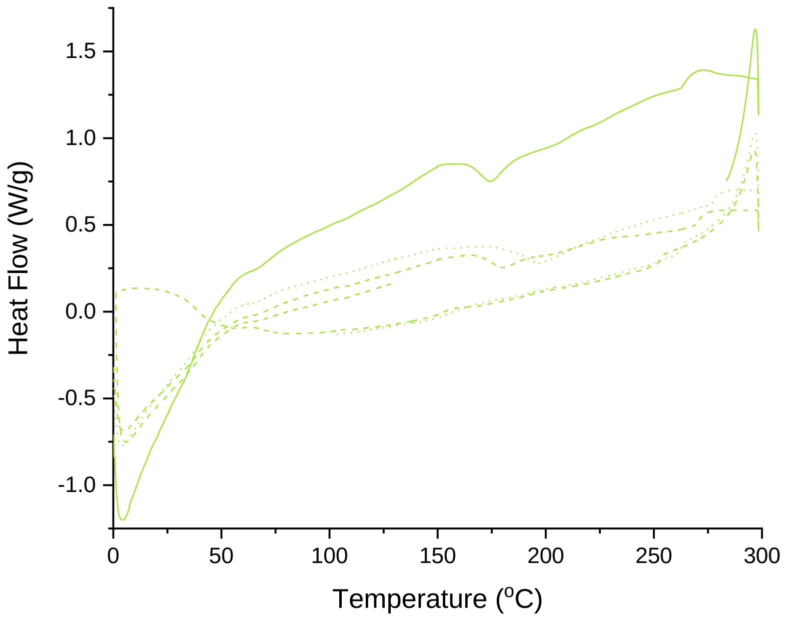

Figure S12: DSC plots for [6FDA I4A pXy][Tf_2N]: 2 eq. [C_4mim][Tf_2N].

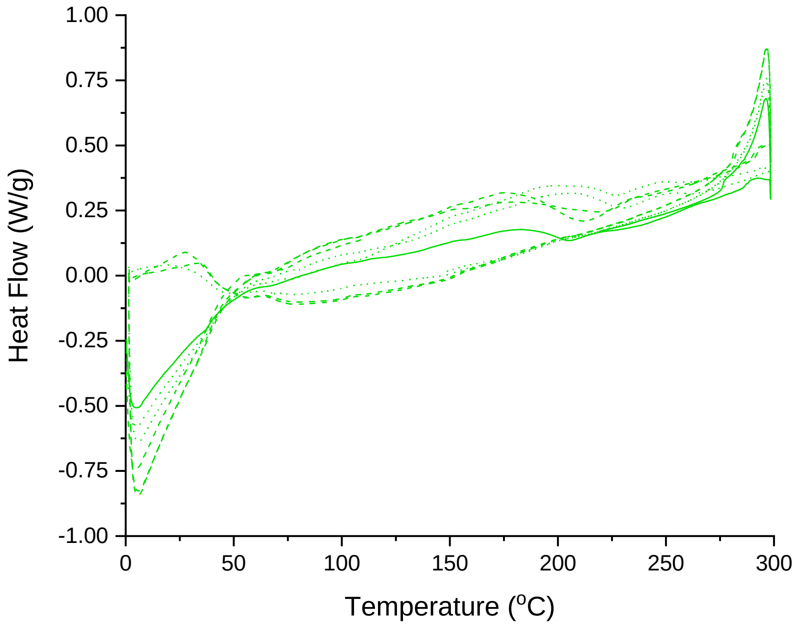

Figure S13: DSC plots for [6FDA I4A pXy][Tf_2N]: 2 eq. [Bnmim][Tf_2N].

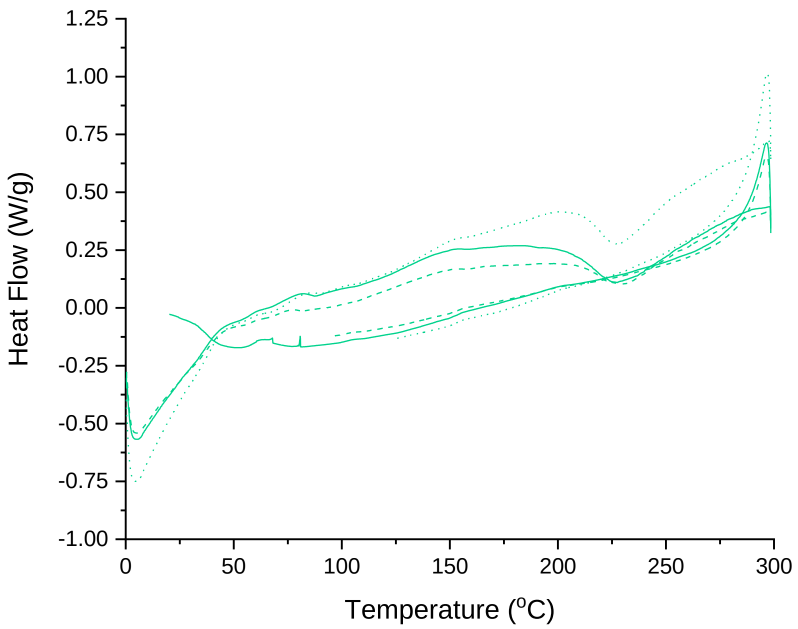

Figure S14: DSC plots for [6FDA I3A mXy][Tf_2N] Neat.

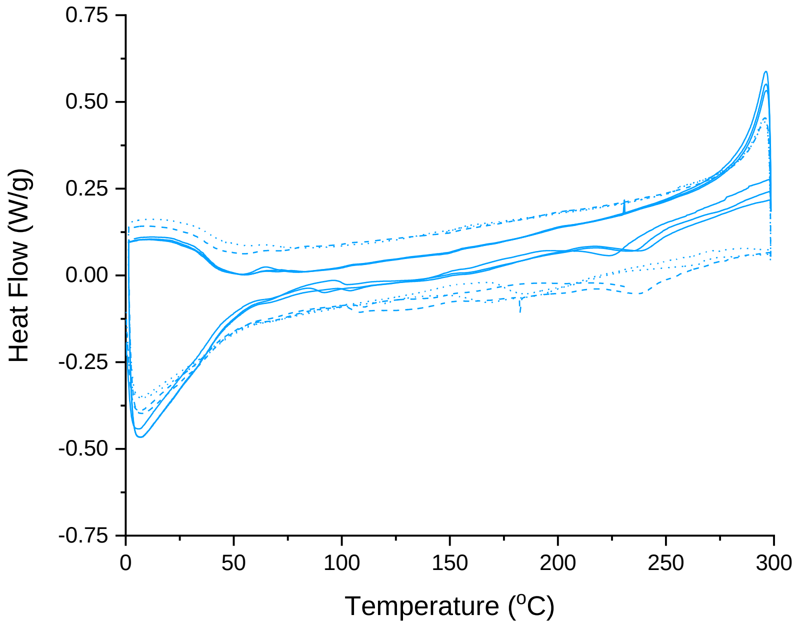

Figure S15: DSC plots for [6FDA I3A mXy][Tf_2N]: 2 eq. [C_2mim][Tf_2N].

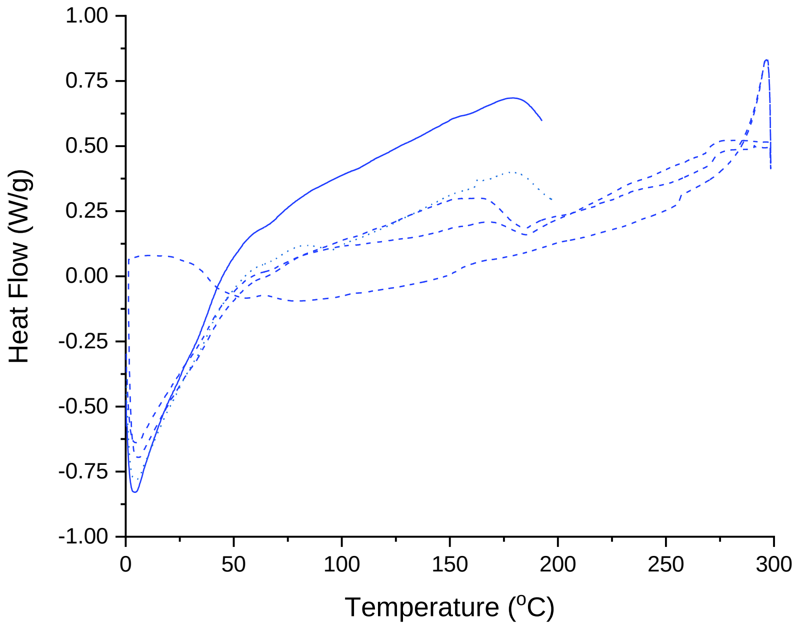

Figure S16: DSC plots for [6FDA I3A mXy][Tf_2N]: 2 eq. [C_4mim][Tf_2N].

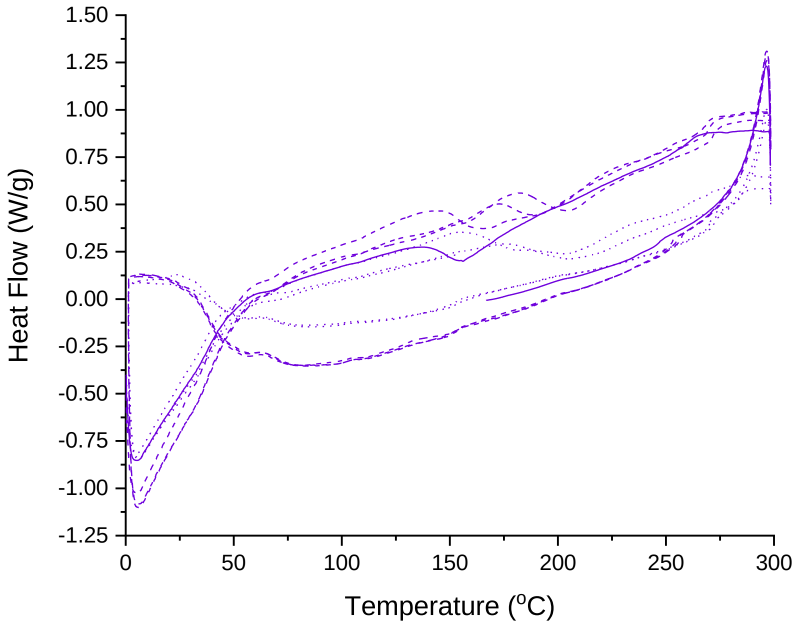

Figure S17: DSC plots for [6FDA I3A mXy][Tf_2N]: 2 eq. [Bnmim][Tf_2N].

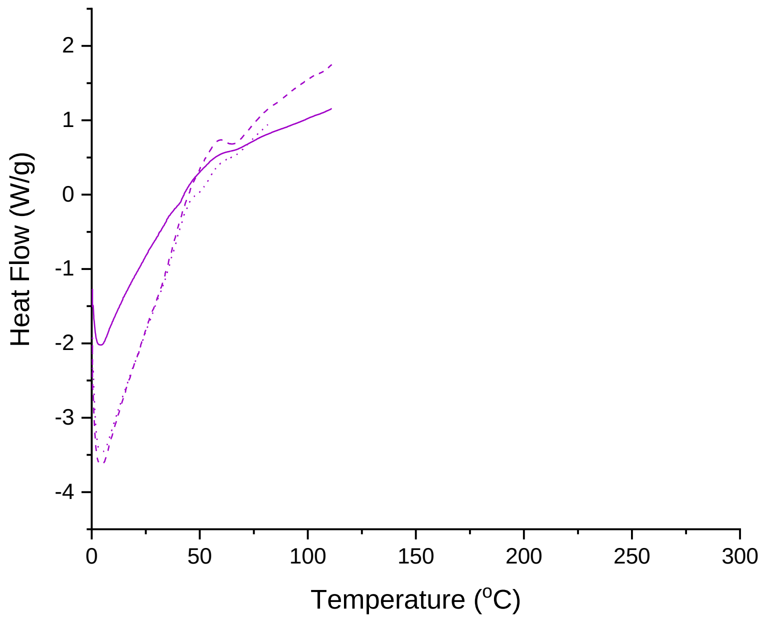

Figure S18: DSC plots for [6FDA I2A oXy][Tf_2N] Neat.

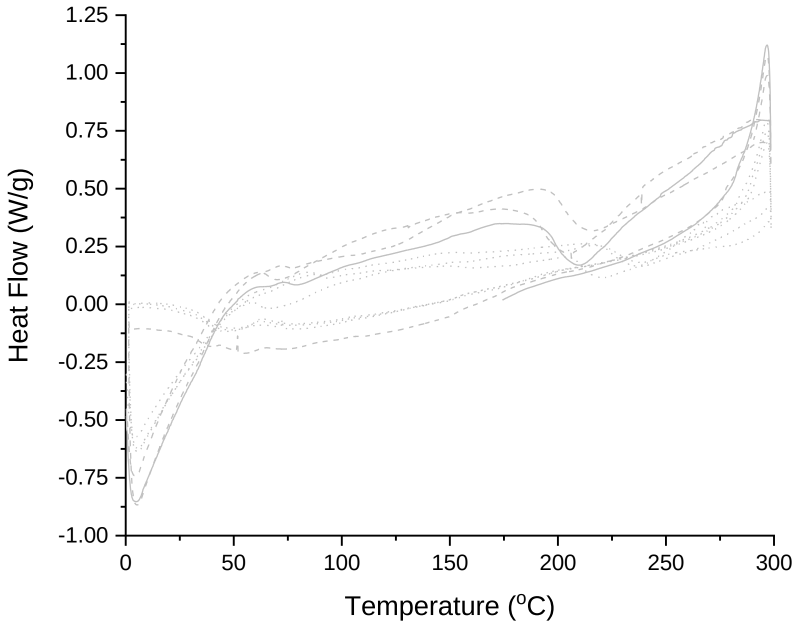

Figure S19: DSC plots for [6FDA I2A oXy][Tf_2N]: 2 eq. [C_2mim][Tf_2N].

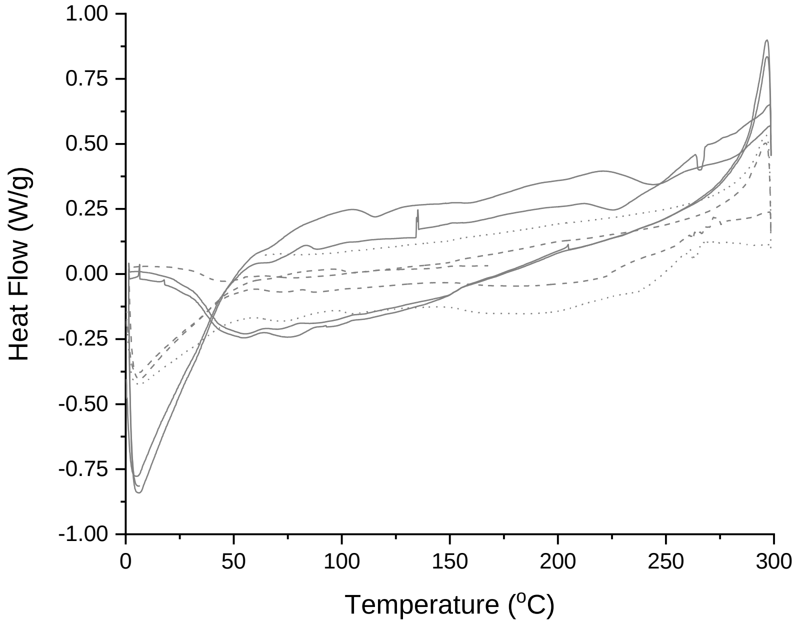

Figure S20: DSC plots for [6FDA I2A oXy][Tf_2N]: 2 eq. [C_4mim][Tf_2N].

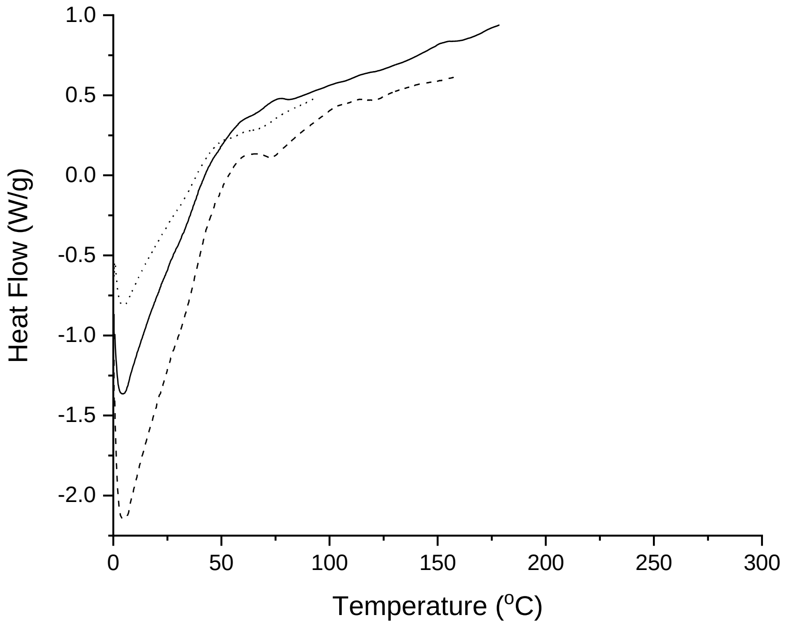

Figure S21: DSC plots for [6FDA I2A oXy][Tf_2N]: 2 eq. [Bnmim][Tf_2N].

Mass Spectroscopy

The number average molecular weight (M_N) was determined via MALDI-TOF mass spectrometry. Each neat polyimide ionene was dissolved in DMAc or DMF and deposited on the plate, with BSA protein in MeOH as the standard for instrument calibration.

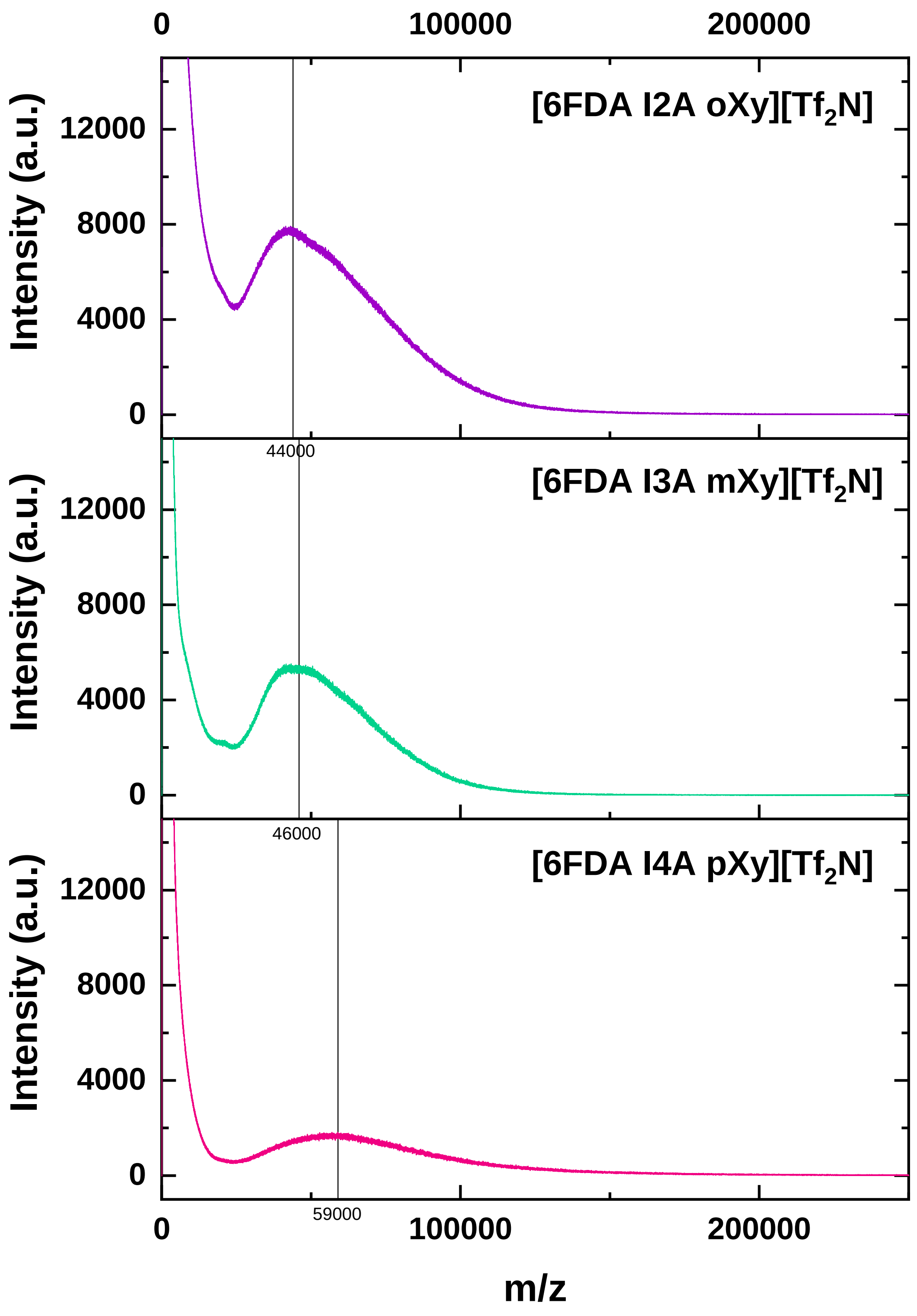

Figure S22: MALDI-TOF spectra for the three, neat polyimide-ionenes.

High Resolution Mass Spectrometry: 6FDA I3A & 6FDA I2A

6FDA I3A

Mass = 726.1439

Calc. Mass = 726.1450

mDa = -1.1; ppm = -1.5

$C_{37} H_{20} N_6 O_4 F_6$

6FDA I2A

Mass = 726.1443

Calc. Mass = 726.1450

mDa = -0.7; ppm = -1.0

$C_{37} H_{20} N_6 O_4 F_6$

X-Ray Diffraction Data

See below for the X-ray diffraction patterns for derivatives **1-14**. The corresponding dspacing values are included in the main manuscript.

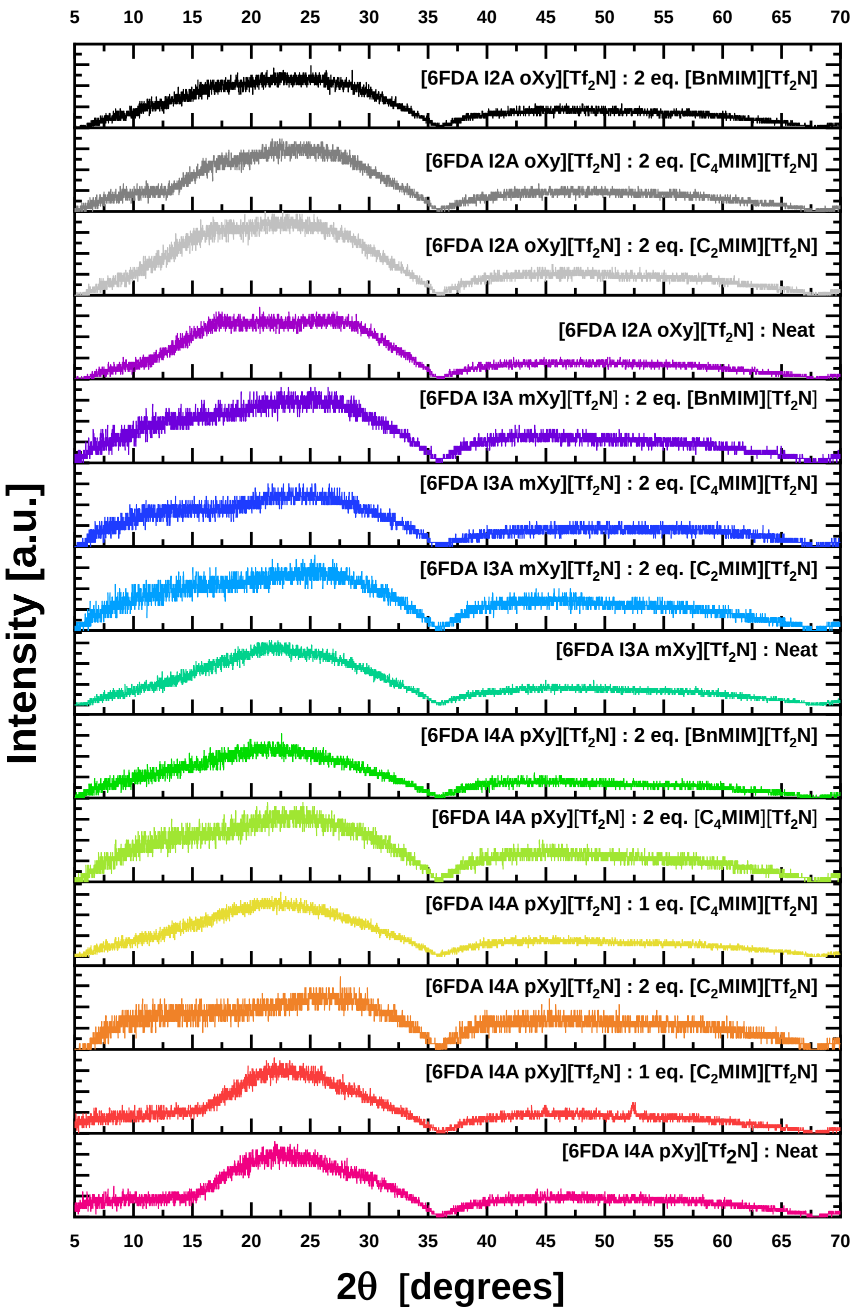

Figure S23: XRD spectra for all samples, with 2θ values from 5-70 °.

Scanning Electron Microscopy (SEM) Images

Images were captured on a Thermo Scientific Apreo FE-SEM. Membranes (1-4) were solvent cast, then fractured in liquid N_2. The cross-section of derivatives 1-4 was captured at magnifications between 500X-1000X.

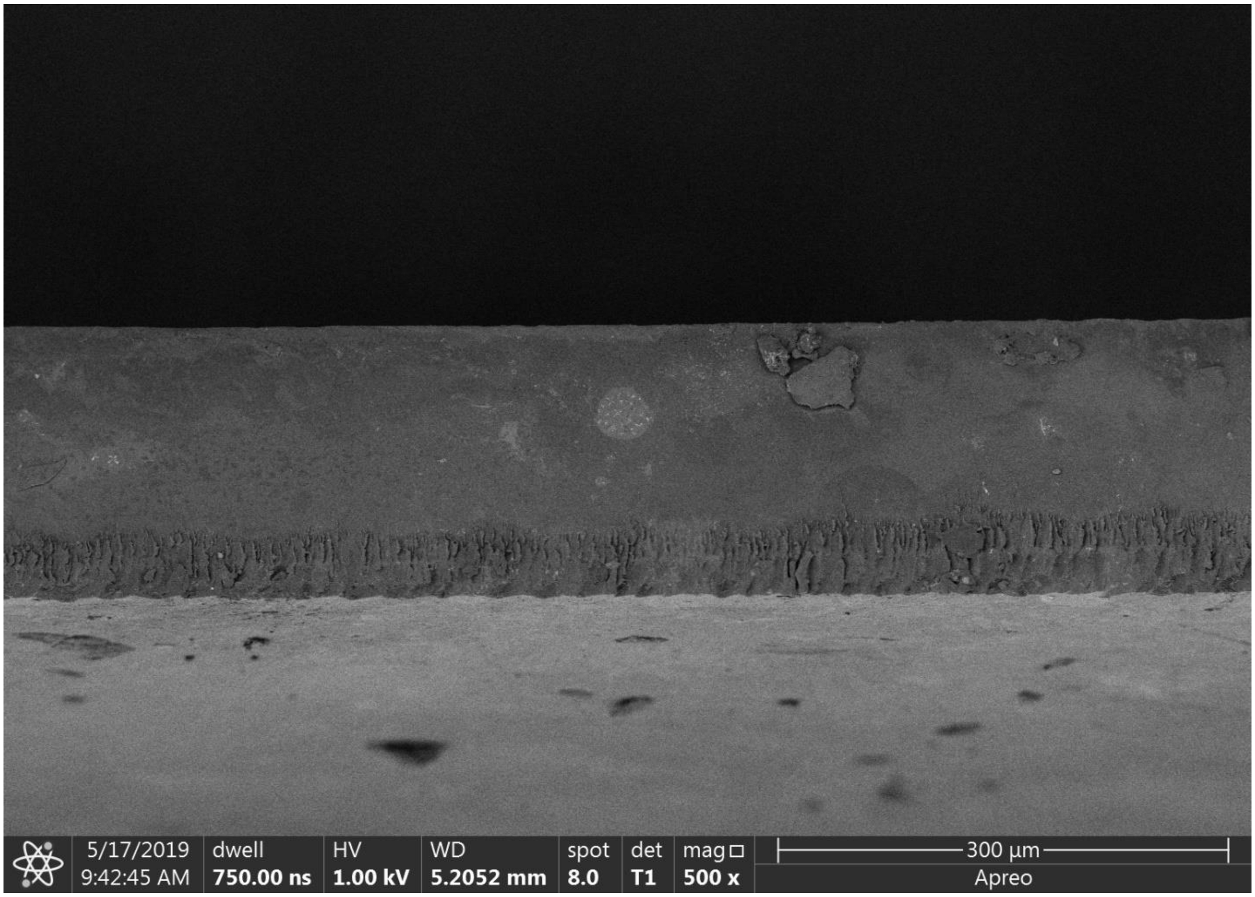

Figure S24: SEM image of [6FDA I4A pXy][Tf_2N] (Neat).

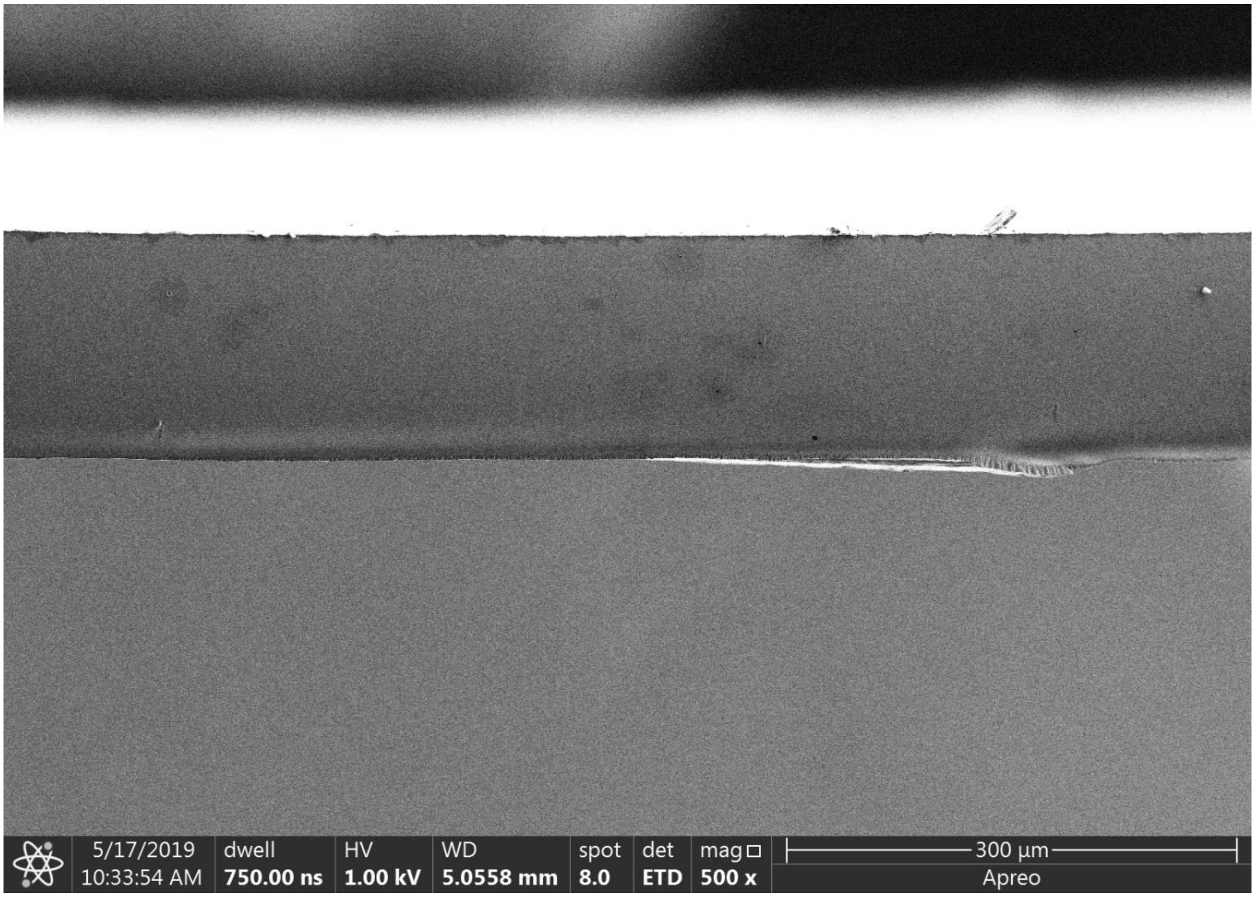

Figure S25: SEM image of [6FDA I4A pXy][Tf_2N] : [C_2mim][Tf_2N] (1 equivalent).

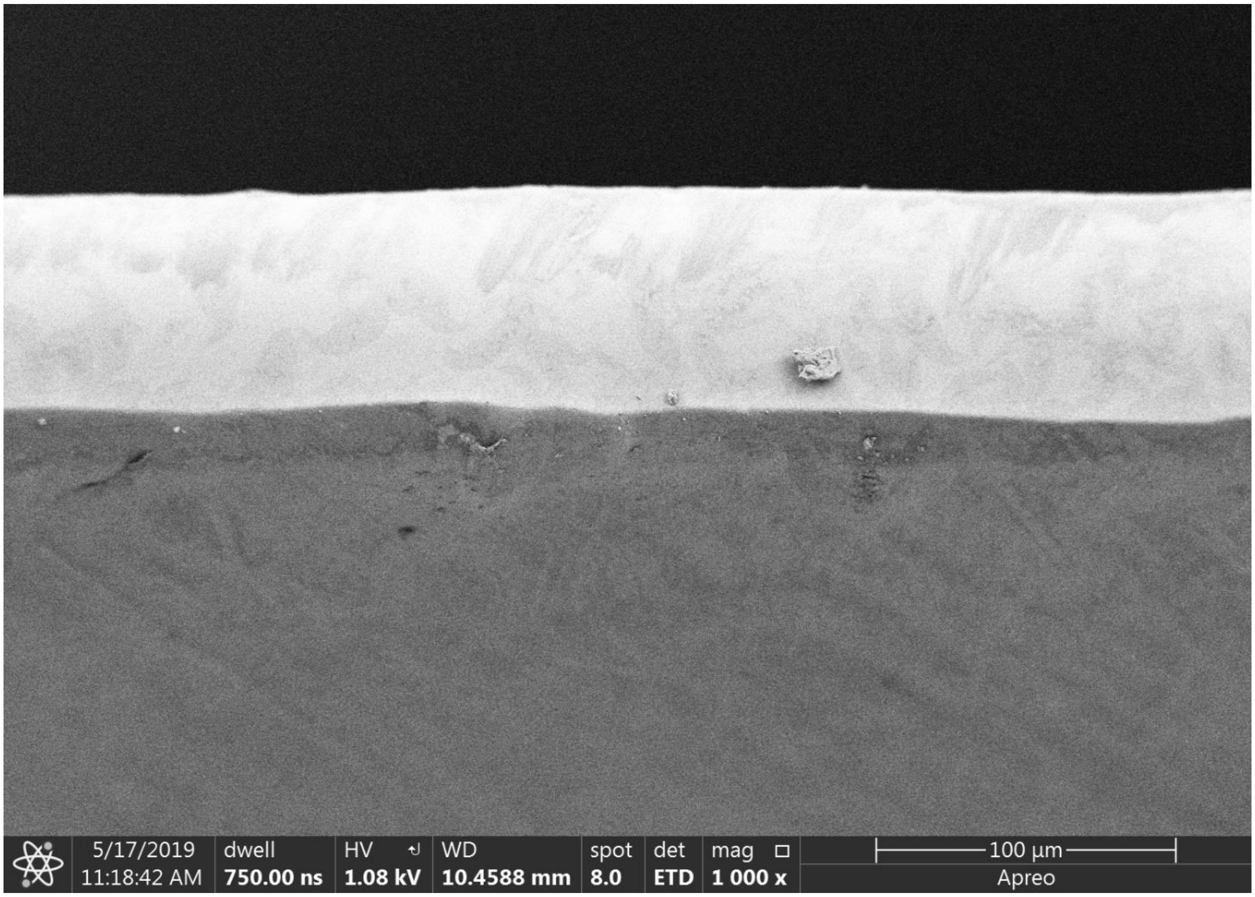

Figure S26: SEM image of [6FDA I4A pXy][Tf_2N] : [C_2mim][Tf_2N] (2 equivalents).

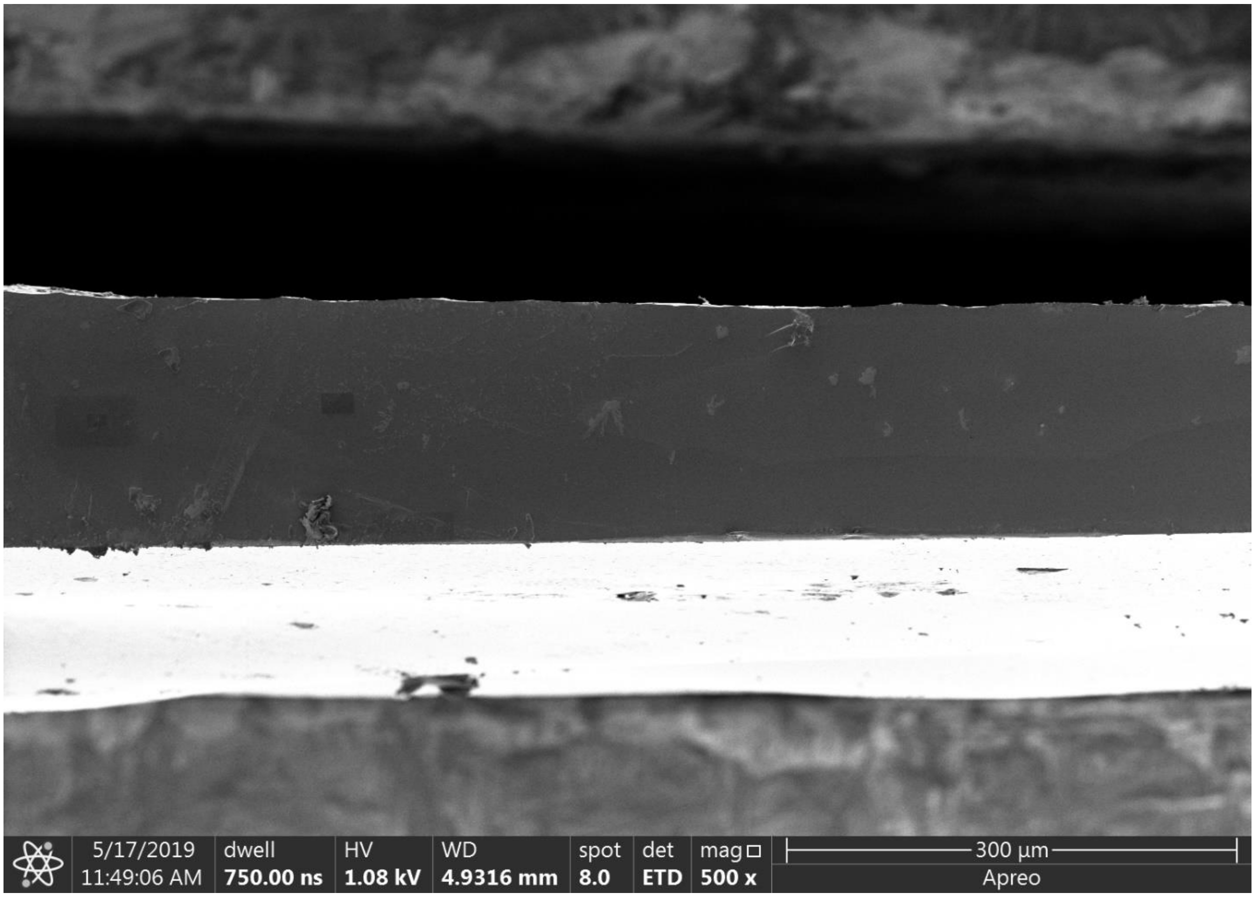

Figure S27: SEM image of [6FDA I4A pXy][Tf_2N] : [C_4mim][Tf_2N] (1 equivalent).
